# Biosynthesis of the corallorazines, a widespread class of antibiotic cyclic lipodipeptides†

**DOI:** 10.1039/d4cb00157e

**Published:** 2024-08-16

**Authors:** Teresa M. Dreckmann, Lisa Fritz, Christian F. Kaiser, Sarah M. Bouhired, Daniel A. Wirtz, Marvin Rausch, Anna Müller, Tanja Schneider, Gabriele M. König, Max Crüsemann

**Affiliations:** a Institute of Pharmaceutical Biology, University of Bonn Nussallee 6 53115 Bonn Germany cruesemann@uni-bonn.de; b Institute for Pharmaceutical Microbiology, University Hospital Bonn, University of Bonn Meckenheimer Allee 168 53115 Bonn Germany; c German Center for Infection Research (DZIF), Partner Site Bonn-Cologne Bonn Germany

## Abstract

Corallorazines are cyclic lipodipeptide natural products produced by the myxobacterium *Corallococcus coralloides* B035. To decipher the basis of corallorazine biosynthesis, the corallorazine nonribosomal peptide synthetase (NRPS) biosynthetic gene cluster *crz* was identified and analyzed in detail. Here, we present a model of corallorazine biosynthesis, supported by bioinformatic analyses and *in vitro* investigations on the bimodular NRPS synthesizing the corallorazine core. Corallorazine biosynthesis shows several distinct features, such as the presence of a dehydrating condensation domain, and a unique split adenylation domain on two open reading frames. Using an alternative fatty acyl starter unit, the first steps of corallorazine biosynthesis were characterized *in vitro*, supporting our biosynthetic model. The dehydrating condensation domain was bioinformatically analyzed in detail and compared to other modifying C domains, revealing unreported specific sequence motives for this domain subfamily. Using global bioinformatics analyses, we show that the *crz* gene cluster family is widespread among bacteria and encodes notable chemical diversity. Corallorazine A displays moderate antimicrobial activity against selected Gram-positive and Gram-negative bacteria. Mode of action studies comprising whole cell analysis and *in vitro* test systems revealed that corallorazine A inhibits bacterial transcription by targeting the DNA-dependent RNA polymerase.

## Abstract 2

Corallorazines are cyclic lipodipeptide natural products produced by the myxobacterium *Corallococcus coralloides* B035. Corallorazine biosynthesis was analyzed *in vitro* and *in silico*, in addition to antibiotic and mode of action studies.

## Graphical abstract

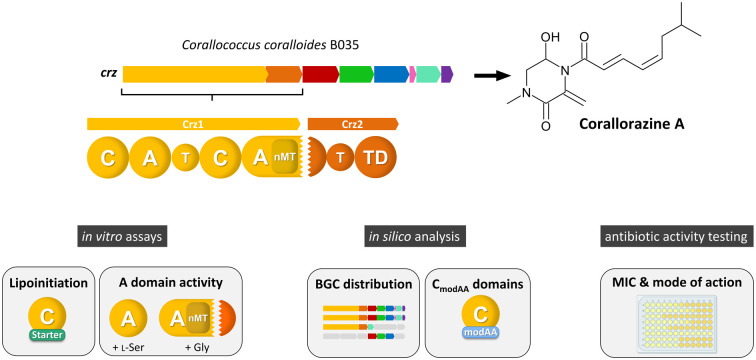

## Introduction

Soil-dwelling myxobacteria (Myxococcales) have been proven to be prolific producers of bioactive specialized metabolites with promise for pharmaceutical applications.^1,2^ Their highly developed secondary metabolism allows them to orchestrate a complex and unusual lifestyle: solitary cells aggregate into a collective preying swarm, with individual cells adopting different morphologies. In nutrient-deficient conditions, these cells form fruiting bodies, where central cells become persistent myxospores.^3^ Myxobacteria have large genomes of up to 16 Mbp, that contain large numbers of biosynthetic gene clusters (BGCs), encoding the biosynthesis of various bioactive, specialized metabolites. Many of these are still unexplored, making myxobacteria a popular object of natural product research.^1^

**Fig. 1:**
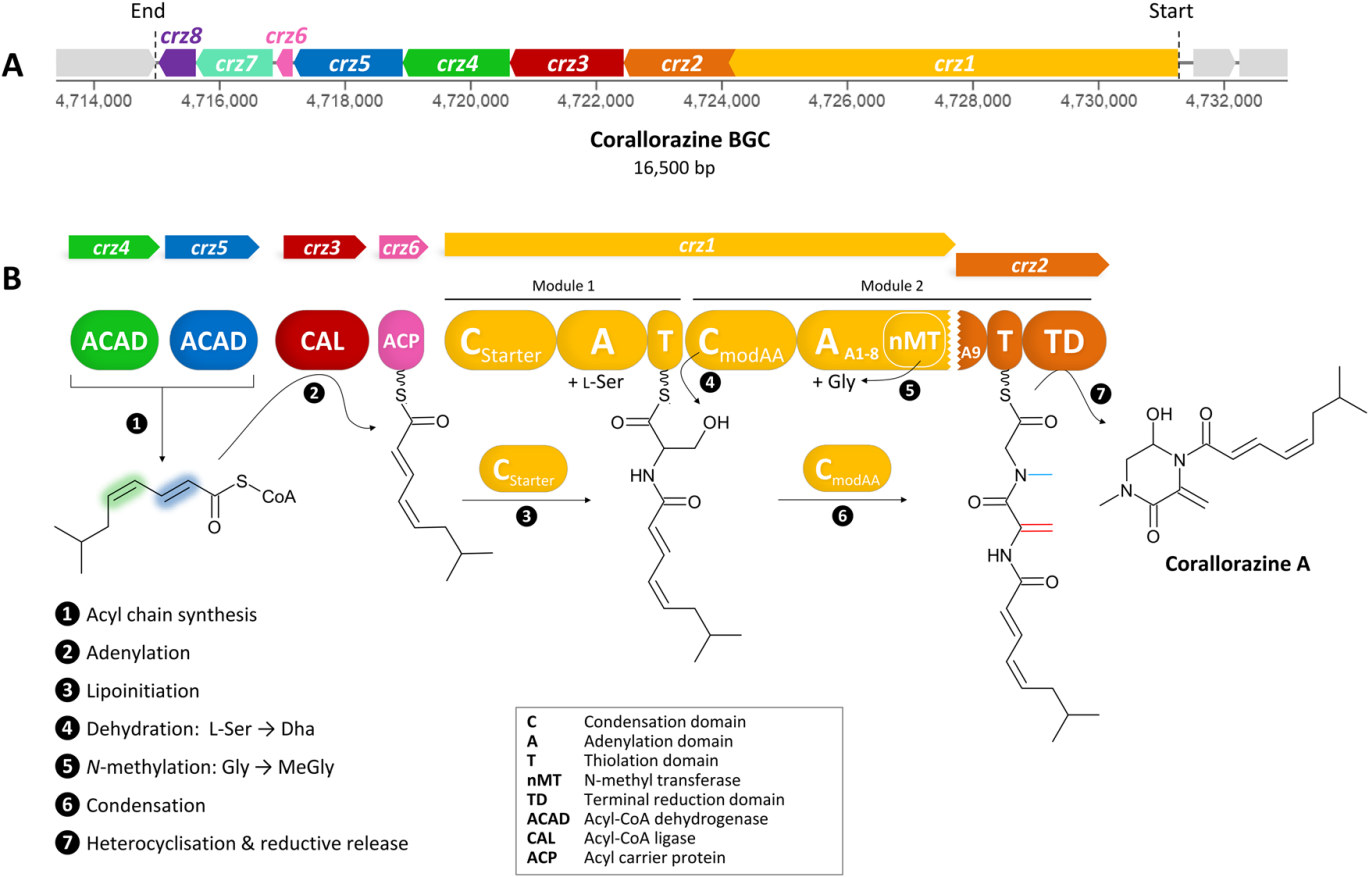
(A) The *crz* BGC. (B) Model of corallorazine A–C biosynthesis. The double bond resulting from dehydration of l-serine is marked in red, the *N*-methyl group resulting from SAM-dependent methylation of glycine is marked in blue. Abbreviations are explained in the box. A more detailed model of corallorazine biosynthesis is depicted in Fig. S1 (ESI†).

The myxobacterial strain *Corallococcus coralloides* B035, isolated from a Belgian soil sample, is known to synthesize the polyketide/nonribosomal peptide hybrid molecule corallopyronin A,^4^ which is a promising antibiotic in the fight against filariasis.^5,6^ During investigations on the corallopyronins, a novel group of secondary metabolites named corallorazines was isolated from *C. coralloides* B035 (Fig. 1).^7^ The structure of the main metabolite corallorazine A was identified as a dipeptide core, composed of *N*-methylglycine and dehydroalanine (Dha) cyclized *via* a hemiaminal forming a piperazine ring. The Dha residue is additionally acylated with the rare branched fatty acid (2*E*,4*Z*)-iso-octa-2,4-dienoic acid. The less complex derivatives corallorazine B and C were identified as probable intermediates of corallorazine biosynthesis, as both are lacking the glycine residue and the latter compound also the *N*-methyl group. A first investigation into the biosynthetic origin of corallorazine A was carried out by employing feeding experiments with 1-^13^C labeled glycine, leading to a significant enhancement of the respective glycine carbon signal as well as that of the Dha residue in the ^13^C NMR spectrum. The authors thus postulated the initial incorporation of serine, which is linked to glycine biosynthesis by the serine-hydroxymethyl transferase, and a subsequent dehydration event to form the final Dha building block. More recently, we fully sequenced *C. coralloides* B035, allowing us to mine its genome for corallorazine biosynthesis genes.^8^

Here, we report the identification and detailed analysis of the corallorazine BGC. We provide a biosynthetic model and investigate corallorazine biosynthesis by *in vitro* experiments on purified non-ribosomal peptide synthetase (NRPS) multidomain proteins. We perform detailed bioinformatic analyses of the dehydrating C domain Crz1C_2_ and compare it with other modifying C domains. We analyze the distribution of the corallorazine gene cluster family and its encoded chemical diversity. Furthermore, we re-evaluate the antibacterial activity of corallorazine A, revealing activity towards *Staphylococcus aureus* strains and identify RNA polymerase as antibiotic target.

## Results and discussion

### Identification of and bioinformatic analysis of the *crz* BGC

To identify corallorazine biosynthesis genes, the genome sequence of *C. coralloides* B035 was analyzed by the bioinformatic pipeline antiSMASH 7.1.0,^9^ revealing 37 natural product BGCs. Some of the detected BGCs appear to be superclusters predicted to synthesize more than one set of specialized metabolites. As we anticipated an NRPS being involved in corallorazine A biosynthesis, we focused on three NRPS BGCs and nine NRPS/polyketide synthase hybrid BGCs that were detected. Amongst these, only three contained adenylation (A) domains predicted to activate and incorporate serine and glycine. Out of these three, only one BGC encoded a bimodular NRPS, predicted to be sufficient for corallorazine A biosynthesis.

The supercluster containing the candidate NRPS genes comprises 38 open reading frames (ORFs), spanning over 87,792 kb. We assigned eight of these, namely *crz1*–*crz8*, to be putatively involved in the biosynthesis of corallorazine A (Fig. 1(A) and Table S1, ESI†). The core biosynthetic genes *crz1* and *crz2* encode for a bimodular NRPS system (Fig. 1(B)). The encoded megaenzyme Crz1 has the domain sequence C_1_-A_1_-T_1_-C_2_-A_2_-nMT, suggesting that the second module is split across two ORFs, as its thiolation (T) domain is absent. A preliminary condensation (C) domain analysis with representative sequences retrieved from NaPDoS^10^ revealed that C_1_ clades with C_Starter_ domains, catalyzing *N*-acylation of the adjacent T domain-bound amino acids, whereas C_2_ is classified as a C_modAA_ domain. Members of this domain subfamily have been long postulated to catalyze dehydration of carrier protein-bound serine and threonine to yield Dha and dehydrobutyrine, respectively.^10^ This hypothesis was recently experimentally confirmed for the biosyntheses of albopeptide^11^ and the antibiotic 2-amino-4-methoxy-*trans*-3-butenoic acid,^12^ in the latter case followed by structural characterization of the dehydrating domain. The proposed substrate specificities of the Crz A domains A_1_ and A_2_ were l-Ser and Gly. The A_2_ domain additionally harbours a type I SAM-dependent *N*-methyltransferase (nMT) domain.^13^ The much smaller ORF *crz2* only encodes the T domain of the second module, as well as a terminating thioreductase (TD) domain, which is known to catalyse the reductive release and cyclisation of nonribosomal peptides at the expense of NAD(P)H.^14^ Taken together, all these features align well with formation of the dipeptidic Dha-*N*-methyl-Gly core of corallorazine A.

The genes *crz3*, *crz4*, *crz5*, and *crz6* encode enzymes that are likely involved in the formation of the unusual acyl residue (2*E*,4*Z*)-iso-octa-2,4-dienoic acid. This residue shows significant similarity to branched, dehydrated fatty acid residues found in several lipopeptide antibiotics, such as cadaside, ramoplanin, friulimicin, laspartomycin, and malacidin.^15,16^ The substrates for its biosynthesis likely originate from fatty acid metabolism through the condensation of isovaleryl-CoA with two malonyl-CoA units. The genes *crz4* and *crz5* encode acyl-CoA dehydrogenases, predicted to introduce double bonds in the acyl precursor. Crz4 is predicted to use isovaleryl-CoA as a substrate, while no specific substrate could be predicted for Crz5. In the next step, the acyl-CoA ligase Crz3 is expected to transfer the CoA-bound acyl chain to the acyl carrier protein Crz6, which would ultimately serve as a substrate for Crz1 C_1_ (Fig. 1(B) and Fig. S1, ESI†). *crz7* and *crz8* encode a cytochrome P450 monooxygenase and the corresponding flavine mononucleotide reductase. P450 monooxygenases are known to catalyse different reactions in NRP biosynthesis, such as β-hydroxylation^17^ and C–C or C–O–C cyclization.^18^ In this case, however, no distinct function could be assigned for Crz7, as the predicted functions for Crz1–6 are sufficient to pose a comprehensive scheme for corallorazine biosynthesis, which is fully depicted in Fig. S1 (ESI†).

### *In vitro* analyses of the corallorazine NRPS

**Fig. 2:**
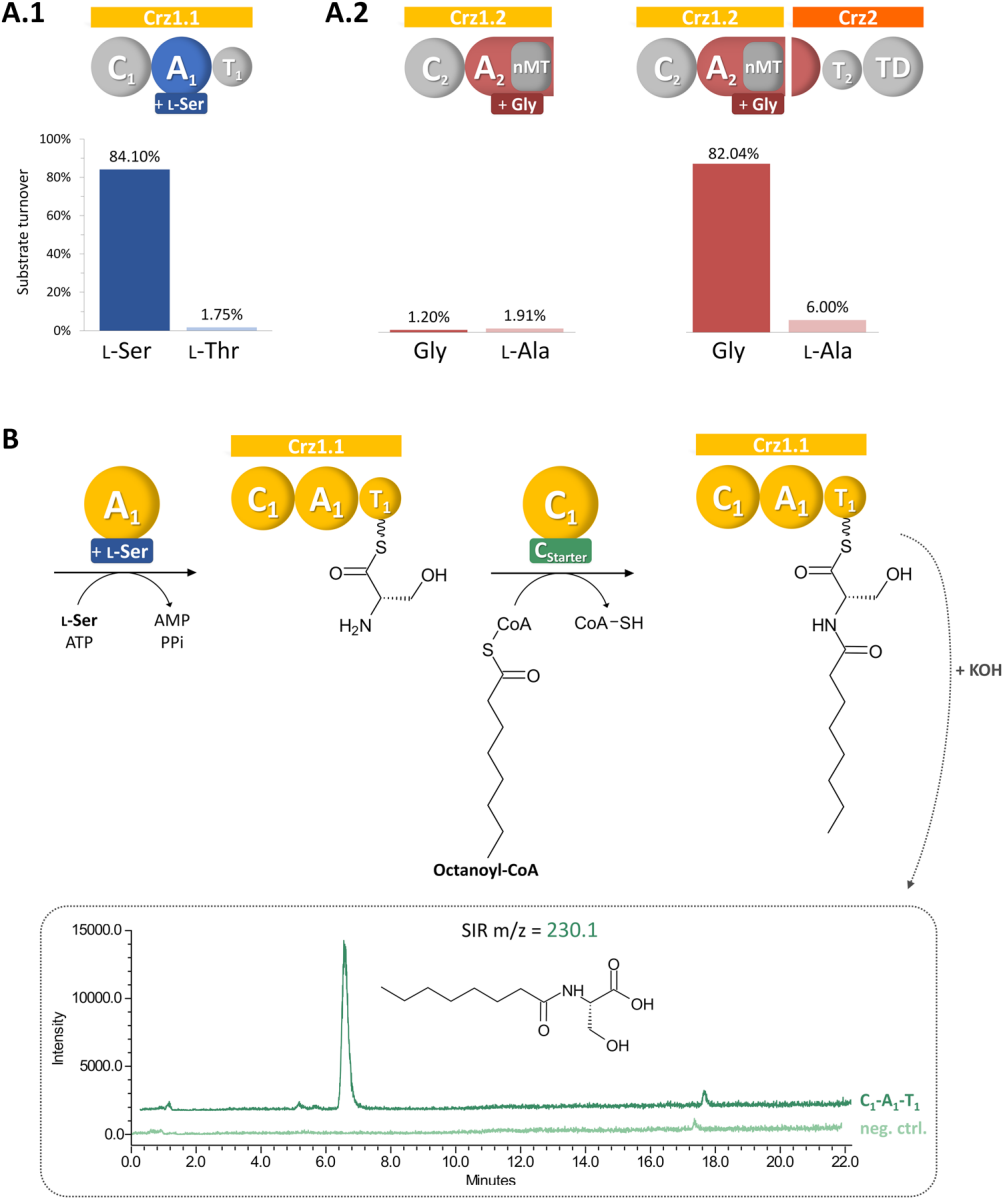
Biochemical characterization of Crz A domains (A) and Crz1-C_1_ (B). Crz A_2_ is only active in presence of domain partly encoded on Crz1 and Crz2 (A.2). Crz1-C_1_ accepts the alternative substrate octanoyl-CoA, leading to the formation of the biosynthetic intermediate octanoylserine, which is detected in LCMS after hydrolysis in single ion recording (SIR) mode.

We aimed to verify our biosynthetic hypothesis for corallorazine by *in vitro* reconstitution of single catalytic steps. To this end, first the larger NRPS gene *crz1* was cloned and heterologously expressed in *E. coli*. The 7,131 bp gene was separated into two expression constructs, Crz1.1 (containing C_1_-A_1_-T_1_) and Crz1.2 (C_2_-A_2_-MT) to facilitate the isolated assessment of A and C domain activity, enable efficient expression, and purification. The constructs were cloned into pET28a, expressed in *E. coli* BAP1 with an N-terminal hexahistidinyl tag and purified *via* metal affinity chromatography (Fig. S2, ESI†). In a first step, the substrate specificities of the A domains of Crz1.1 and Crz1.2 were analyzed by the γ^18^O_4_-ATP exchange assay.^19^ We observed specific turnover of l-Ser by Crz1.1, which corroborated the predicted biosynthetic origin of Dha (Fig. 2(A)). However, for Crz1.2, no turnover was observed in presence of Gly. A more detailed examination of the A_2_ domain's primary structure revealed that the nMT domain is integrated into the A domain right after core motif A8 and terminates with a stop codon, rendering A_2_ on Crz1 inactive. The missing motives A9 and A10 were instead found to be encoded at the beginning of the adjacent ORF *crz2* (Fig. S3, ESI†). Thus, we also cloned and heterologously expressed *crz2* analogously to the Crz1 constructs. The resulting protein Crz2 (Fig. S2, ESI†) was mixed with Crz1.2 in a 1 : 1 molar ratio and then assessed for adenylating activity with glycine as substrate. Strikingly, in this setup, we observed an almost quantitative substrate turnover in the γ^18^O_4_-ATP exchange assay. To our knowledge, this is the first experimental report of a split, yet functional A domain, encoded on two different genes. This unusual architecture might be the consequence of an integration of the nMT domain or an A subdomain recombination event during evolution, which both were shown to predominantly occur between the motifs A8 and A9.^20^

Next, we conducted a C_Starter_ domain assay, that had previously been established in our laboratory,^21^ to test the activity of Crz1-C1. Due to the unavailability of its proposed substrate, (2*E*,4*Z*)-iso-octa-2,4-dienoic acid, which is predicted to be bound to acyl carrier protein Crz6, we used octanoyl-CoA as alternative substrate together with l-Ser, and ATP for Crz1.1. Indeed, the activated fatty acid was accepted and we could subsequently detect the formation of octanoyl-serine in the assay (Fig. 2(B)), confirming the proposed function of the Crz-C_1_ starter domain and offering the possibility for the targeted generation of corallorazine analogues with altered fatty acid tails.

### Bioinformatic analyses of Crz1-C_2_ reveal additional features of modifying C domains

We next performed a bioinformatic analysis of Crz1-C_2_, which was predicted to be a dehydrating C domain belonging to the C_modAA_ clade.^10^ First homologues of this clade were only recently characterized experimentally.^11,12^ We first generated a phylogenetic tree including the two C domains of the Crz NRPS and representative C domains of the individual subclasses based on the sequence set of Wheadon and Townsend.^22^ To ensure a broad taxonomic coverage and to include additional known subtypes in the analysis, the set was extended with selected C domains from further biosynthetic pathways to yield a total of 275 C domains. The resulting tree is in good agreement with other studies and split into three branches (Fig. S4, ESI†), with an l-clade and a d-clade, representing the C domain family in a narrower sense. In the d-clade, C_modAA_ domains, dual epimerase/condensation (E/C) and ^D^C_L_ domains, condensing d- and l-configured amino acids, each form monophyletic groups.

**Fig. 3:**
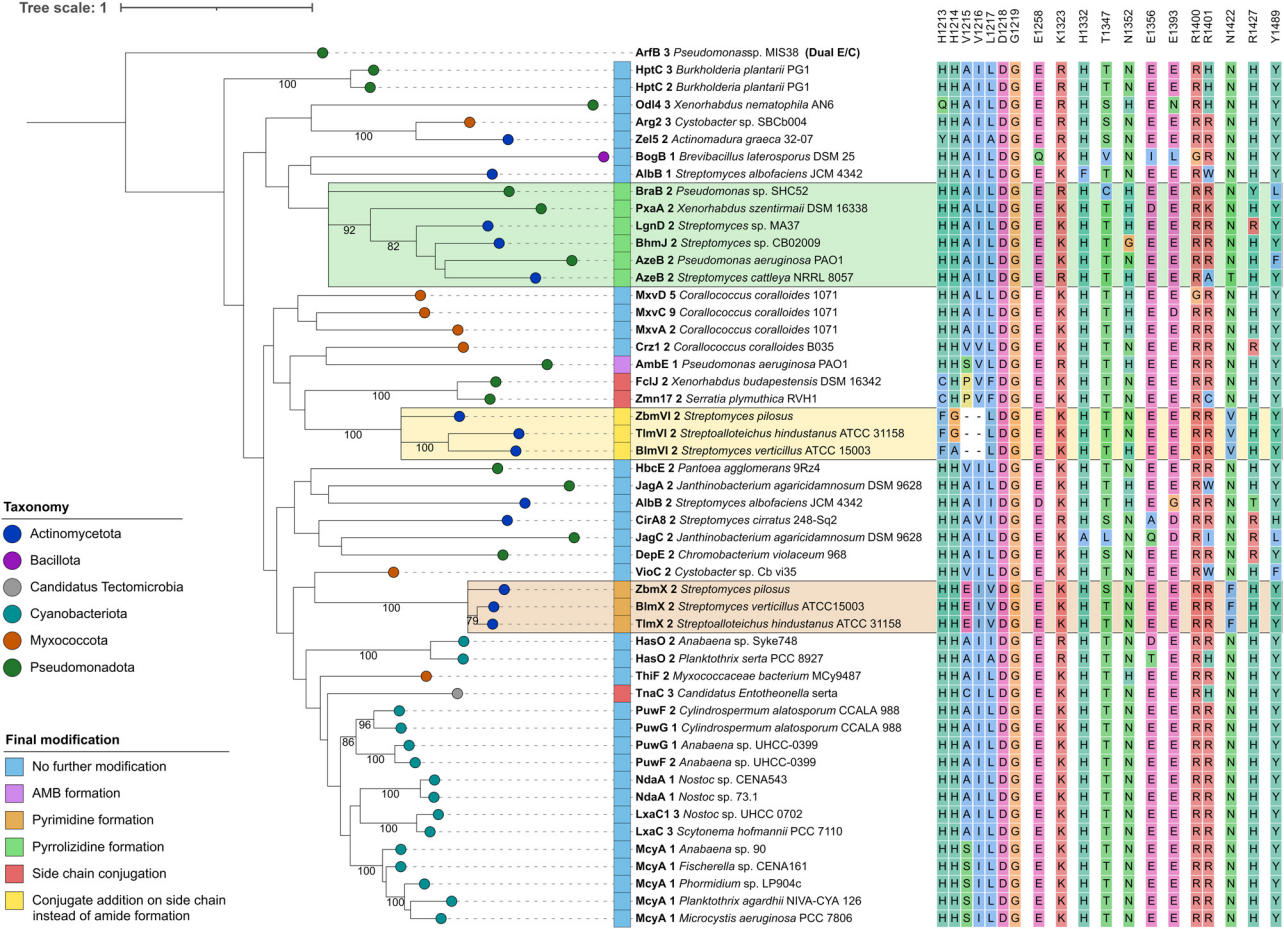
Phylogenetic analysis of C_modAA_ domains. A maximum likelihood tree of 50 C_modAA_ domains was generated using one randomly selected dual E/C domain as outgroup. A total of 100 bootstrap replicates were performed and bootstrap values above 75 are given on corresponding branches. Domains were assigned colours according to the taxonomic classification of the source organism at phylum level, and by final modification of the amino acid they act on. Conserved residues detected in this study are depicted next to each domain. Visualisation was done with iTol v6.^24^

C_modAA_ domains can further be distinguished by the final modification of their substrate amino acid in addition to simple dehydration. In a previous study, 27 domains from the C_modAA_ subtype were identified and analysed with regard to their type of modification.^12^ We aimed to get a more comprehensive picture and expanded this set with 23 additional sequences of potential C_modAA_ domains, including Crz1-C_2_, that were identified by a combination of literature and MIBiG^23^ analyses (Table S2, ESI†). The resulting phylogenetic tree (Fig. 3) shows that domains catalyzing conjugate addition instead of peptide bond formation and pyrimidine formation each formed their own clades. The pyrrolizidine formation group, which was now extended to six domains, also formed its own clade, while Crz1-C_2_ was repeatedly grouped with AmbE C1.

In order to identify conserved amino acid residues and motifs within C_modAA_ domains, potentially offering clues to their mode of action, the alignment (Fig. S5 and S6, ESI†) was further investigated and also compared to ^D^C_L_ and dual E/C domain sequences, related subclasses within the d-clade (Fig. S7, ESI†). Previously, several well conserved residues located near the active centre or the donor side tunnel of AmbE-C_1_ were identified.^12^ For example, mutagenesis of active site residues R1605 and H1632 significantly reduced the activity of AmbE-C_2_, indicating their importance for the function of C_modAA_ domains. In our alignment, R1605 (K1325 in Crz1-C_2_), that was predicted to take part in substrate positioning,^12^ shows functional conservation in all analysed C_modAA_ domains (78% K, 22% R). Several other residues were found to be highly to moderately conserved among all C_modAA_ domains in the alignment (Fig. 3). Noticeably, some of these were located in inter-motif regions, and partly arranged in motif-like sections that were not present in this form in any of the other representatives of the d-clade (Fig. S7, ESI†). Particularly striking was a section directly located N-terminal of the C5 motif, which extends over approx. 8 amino acids and, in a structural model generated with AlphaFold, is part of a helix on the donor side of the substrate tunnel (Fig. S8 and S9, ESI†).

This helix contains the highly conserved K/R mentioned above, but also a noteworthy histidine (H1332), which is present in 48 of the 50 analyzed C_modAA_ domains. In our structural model, the side chain of H1332 is oriented in the same direction as that of K1325 (Fig. S9, ESI†). Together, K1325 and H1332 seem to delineate the beginning and end of a motif-like region characterized by the consensus sequence [K/R]SVLLAAH, which is, in the model structure, in close proximity to conserved T (T1347, 82%), E (E1393, 88%), and Y (Y1489, 90%) side chains.

The HHxxxDG motif in the active site is crucial for the activity of canonical C domains.^25^ Our alignment does show strict conservation of the DG motif in C_modAA_ domains, but not of both histidines. In seven cases, the first His, which is thought be crucial for structural integrity, is missing, while in three other cases the second His is mutated. In FclJ-C_2_ and Zmn17-C_2_, both catalyzing side chain conjugation, the first His is exchanged by a Cys. Given that Cys can also form hydrogen bonds, it might substitute for His in maintaining structural integrity under certain conditions. Interestingly, TnaC-C_3_, the third domain in this group, retains both histidines but features a cysteine directly downstream of the second His. Domains involved in conjugate addition (BlmVI-C_2_, TlmVI-C_2_, and ZbmVI-C_2_) lack both histidines, and feature Phe and Gly or Ala instead. Here too, the presence of Phe could induce an active site conformation which promotes addition to the side chain. The absence of the second His, which is attributed a central role in peptide bond formation, aligns with the altered functions of these domains.

Further specific residues for C_modAA_ domains were located in close proximity to the donor substrate tunnel: these include an R (96%) at the beginning of the C6 motif, followed by a less conserved R (78%), corresponding to R1400/R1401 in Crz1, and R1679/R1680 in AmbE, respectively. The model shows both arginine side chains to be located near the substrate tunnel entrance and are facing the side chain of a conserved E (E1365, 88%) located directly opposite. Mutating R1679 or R1680 to Ala had no significant effect on its activity.^12^ However, it is feasible that one Arg might substitute for the other. In fact, each of the C_modAA_ domains contains either both or at least one of the arginines, which supports their predicted importance for C_modAA_ domain functionality.

### Distribution of the corallorazine BGC

**Fig. 4:**
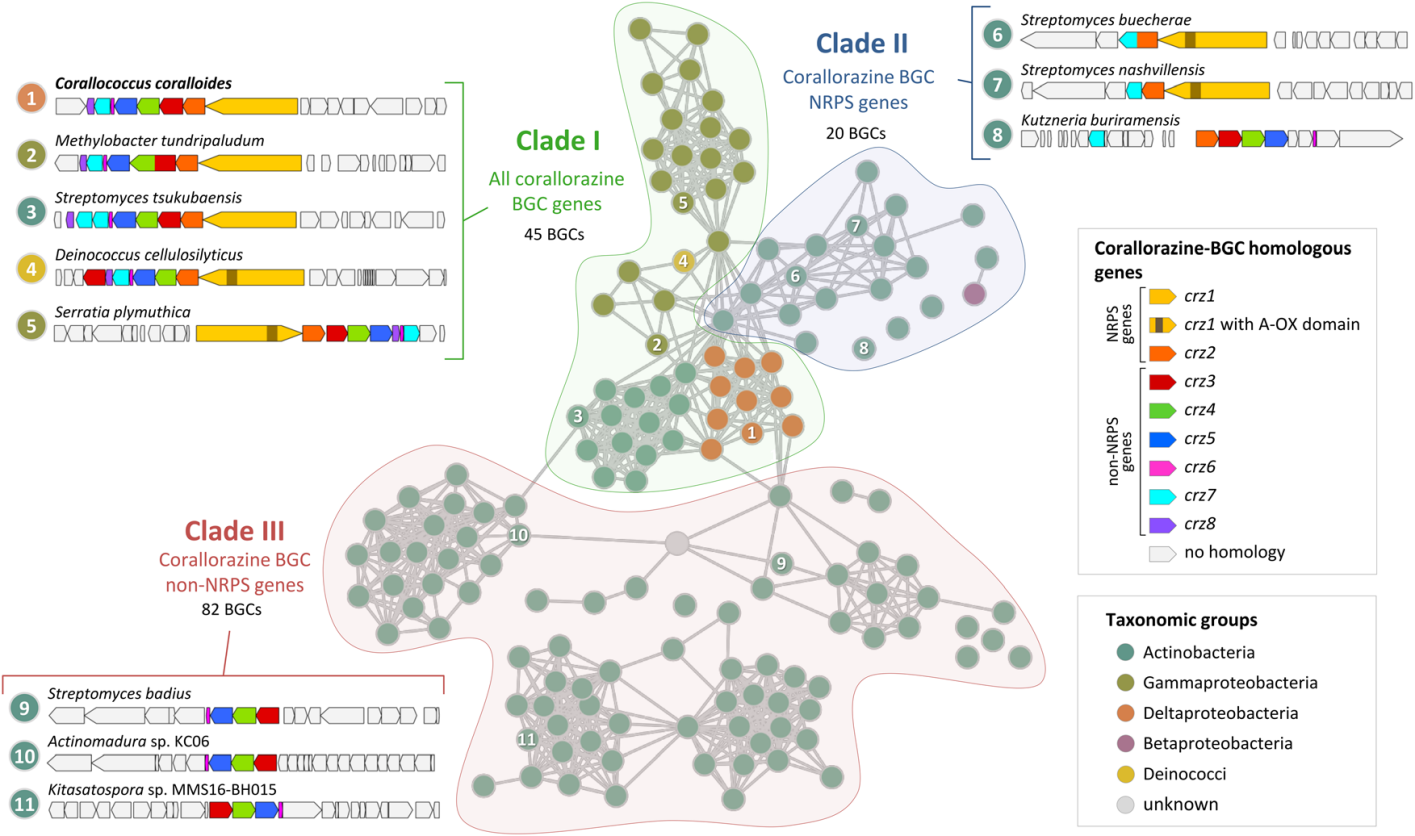
Cytoscape network visualization of homologous corallorazine BGCs. Nodes correspond to BGCs containing at least one gene that is homologous to a gene in the corallorazine BGC (A1, red circle outline). Information on host strain and presence of *crz1–8* homologues is displayed in Table S3 (ESI†). Classification in clade I, II or III is indicated by colored areas. Examples for characteristic clusters from these clades are shown in this figure and in Fig. S11 (ESI†). Length of the connections between nodes correlates with the degree of relationship according to BiG-SCAPE analysis.

During our bioinformatic analyses, we identified multiple highly similar homologues of *crz* genes in the NCBI database. To gain insight into the global distribution and evolution of the *crz* BGC, we extracted all corresponding BGCs from NCBI and submitted them to a network analysis with BiG-SCAPE.^26^ At a cutoff of 0.7, the *crz* BGC clusters with 146 other BGCs, which originate from diverse bacterial genera, including rare natural product producers such as Deinococcus. Most BGCs of the *crz* GCF cluster were detected in actinomycete bacteria, which however may be biased by the high abundance of sequenced members of this genus in the genome databases. BGCs in the resulting network were roughly divided into three clades (I–III) (Fig. 4, Fig. S10, S11 and Tables S3, S4, ESI†).

Clade I consists of BGCs harbouring homologues of all eight *crz* genes, with *crz7* duplicated in most *Streptomyces* species. The most intriguing additional feature is an additional monooxygenase domain (MOx) within A_2_, detected in 38 pathways, notably all from members of the Gammaproteobacteria genera *Serratia* and *Pseudomonas*. This MOx domain was previously reported from myxothiazol and melithiazol biosynthesis, where it is also encoded within glycine-recruiting A domains.^27^ In the respective pathways, the MOx domain hydroxylates C_α_, leading to the spontaneous dissimilation of an amine due to the unstable N–C_α_ bond. The remaining T domain-bound glyoxylate is subsequently released by the terminating TE domain. The presence of additional MOx domains in *crz*-like BGCs suggests the formation of derivatives with truncated, linear peptide cores by these pathways, similar to the shunt products corallorazine B/C.

Clade II of the network analysis contains BGCs that encode homologues of *crz1* and *crz2*, but mostly lack genes corresponding to *crz3–6*, suggesting production of molecules with a cyclic dipeptide core, but lacking the acyl side chain. Interestingly, genes encoding for a P450 monooxygenase and the associated FMN reductase, corresponding to *crz7* and *crz8*, respectively, are present in almost all BGCs from clade II. In five cases, *e.g.* cluster 6 from S*treptomyces buecherae* (Fig. 4), the P450 is even fused to the NRPS. This constellation implies an important function of the P450 for biosynthesis of this compound class. However, our current biosynthetic model does not involve steps catalysed by Crz7 and Crz8. Instead, these enzymes might also play a role in side chain biosynthesis or self-resistance.^28^ The BGCs of clade III harbour genes corresponding to *crz3–6* that are located in close proximity to genes encoding various NRPSs- and/or polyketide synthases (PKS). This indicates a conserved interplay of the encoded enzymes and the collective horizontal transfer of the corresponding genes, yielding structurally diverse compounds with branched, unsaturated fatty acid side chains, such as the calcium-dependent antibiotics of cadasides, malacidins, and taromycins.^16,29^

### Antibiotic activity and mechanism of action

Finally, we determined the antibacterial activity of corallorazine A against a panel of selected Gram-negative and Gram-positive test strains (Table 1). In our previous study, only the activity against *Bacillus* and *Escherichia coli* was tested,^7^ which prompted us to include other pathogenic bacteria in the bioassays. The compound shows moderate activity against Gram-positive pathogens with the highest activity against methicillin-resistant *Staphylococcus aureus* strain COL (MIC 4–8 μg mL^−1^). Corallorazine A lacks activity against *E. coli*, although the permeable efflux-negative *E. coli* strain MB5746 was susceptible, referring to the outer membrane as a crucial barrier for corallorazine A.^30^

**Table 1:** Activity of corallorazine A against selected bacterial strains. MSSA, methicillin-sensitive *S. aureus*; MRSA, methicillin-resistant *S. aureus*, VISA, vancomycin-intermediate *S. aureus*, VRE, vancomycin-resistant enterococci

Strain	MIC [μg mL^−1^]
*Staphylococcus aureus* SG511 (MSSA)	16
*Staphylococcus aureus* RN4220 (MSSA)	32
*Staphylococcus aureus* HG003 (MSSA)	32
*Staphylococcus aureus* Mu50 (VISA, rifampin^R^)	8
*Staphylococcus aureus* COL (MRSA)	4–8
*Enterococcus faecalis* BM4223 (VRE)	16
*Bacillus subtilis* 168	128
*Escherichia coli* I-112768	>128
*Escherichia coli* MB5746	16

**Fig. 5:**
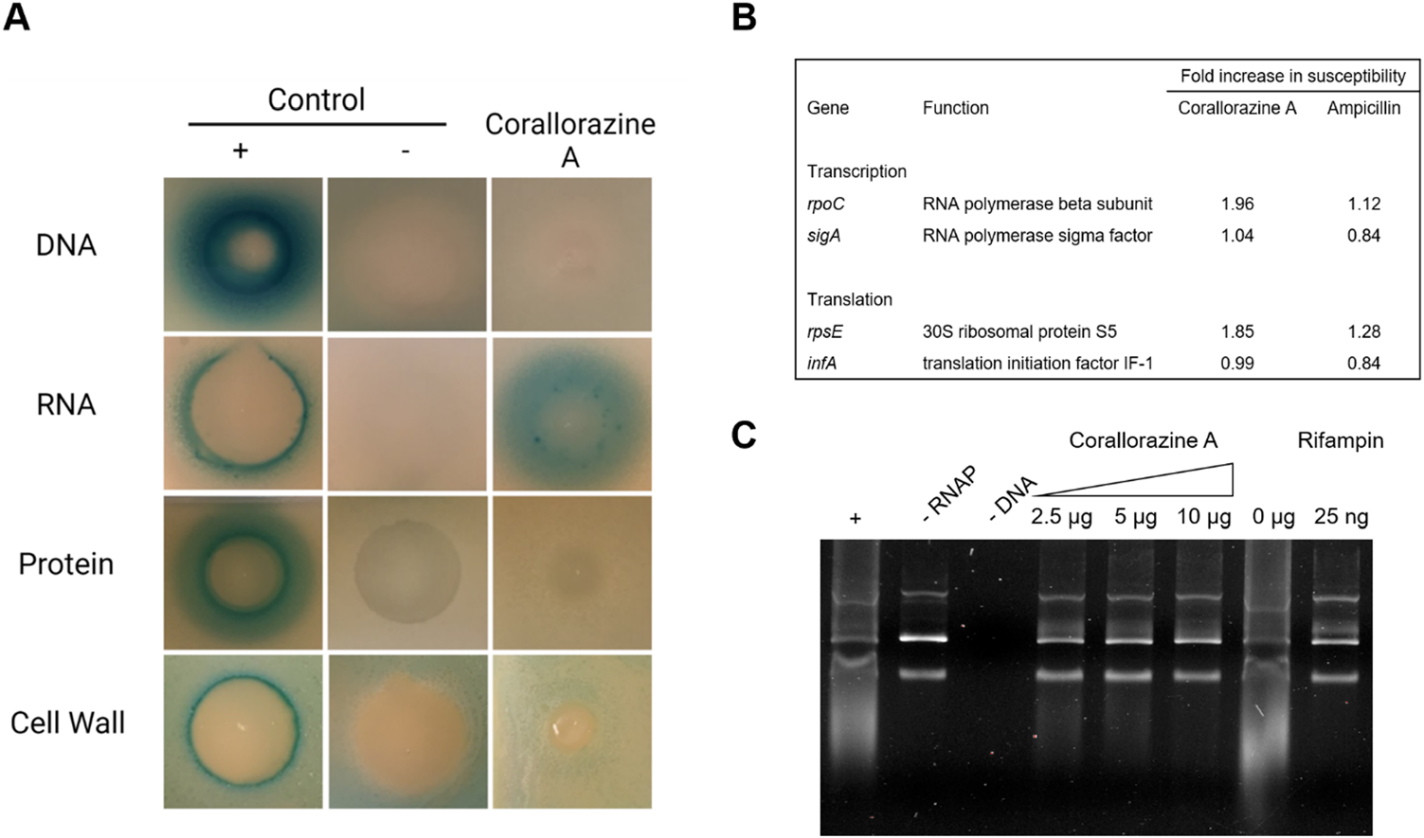
Corallorazine A targets the DNA-dependent RNA polymerase. (A) The effect of corallorazine A on major macromolecular biosyntheses in *B. subtilis*. *B. subtilis* bioreporter strains contain selected promoter-*lacZ* fusions to identify interference with DNA (P_*yorB*_), RNA (P_*yvgS*_), protein (P_*yheI*_), and cell wall (P_*ypuA*_) biosynthesis. A blue halo around the inhibition zone indicates induction of a specific stress response and resulting β-galactosidase expression. The antibiotics ciprofloxacin, rifampicin, clindamycin, and vancomycin served as positive controls. Corallorazine A specifically induces P_*yvgS*_ indicative of interference with RNA biosynthesis. (B) Corallorazine A susceptibility was determined in cells with reduced expression levels of proteins involved in transcription and translation. Expression of *rpoC* and *rpsE* antisense RNA resulted in sensitization towards corallorazine A. The β-lactam antibiotic ampicillin was used as control. (C) Inhibition of RNA polymerase activity by corallorazine A *in vitro*. Agarose gel electrophoresis shows that *E. coli* RNA polymerase *in vitro* transcription activity was reduced in presence of increasing concentrations of corallorazine A.

To determine the target pathway, pathway-selective β-galactosidase bioreporter strains were treated with corallorazine A. Expression of LacZ was specifically induced in *B. subtilis* P_*yvgS*_-*lacZ* indicating interference with RNA biosynthesis while DNA, protein and cell wall biosyntheses remained unaffected (Fig. 5(A)). In a next step, we used conditional antisense RNA expression to reduce expression of putative target proteins involved in transcription and translation to confirm RNA biosynthesis pathway as a target for corallorazine A (Fig. 5(B)). Reduction of target protein results in hypersensitivity towards the inhibitor. While the reduction of expression levels of RNA polymerase Sigma factor SigA (*sigA*) and translation initiation factor IF-1 (*infA*) had no effect, increased susceptibility towards corallorazine A was observed when the RNA polymerase beta subunit (*rpoC*) and the 30S ribosomal protein S5 (*rpsE*) were down-regulated, indicating the latter being molecular targets. The direct effect of corallorazine A on the RNA polymerase from *E. coli* was examined in an *in vitro* transcription assay (Fig. 5(C)). The results show that corallorazine A inhibits transcription in a concentration-dependent manner, suggesting direct interaction with RNA polymerase.

## Conclusions

We identified and analyzed the corallorazine BGC from *C. coralloides* B035 and substantiated our hypothesis for corallorazine biosynthesis by assessing the catalytic activity of single NRPS domains *in vitro*. The respective bimodular NRPS is encoded by two genes *crz1* and *crz2*, whereby the gene borders are within the encoded A domain of the second module. Most parts of this A domain, together with a conventionally embedded *N*-methyltransferase are encoded on *crz1*, whereas the smaller A subdomain, however crucial for catalytic activity, is encoded on *crz2*. This unusual constellation raises questions about its evolutionary origin. In case of an erroneous recombination event, this position would indicate a good starting point for artificial reengineering of NRPS assembly lines.

The minor, linear derivatives corallorazine B and C appear to be biosynthetic shunt products of the assembly line, possibly caused by this unusual split, as both are lacking the glycine residue. In addition, our detailed bioinformatic analyses of the dehydrating Crz1-C_2_ and related C_modAA_ domains revealed previously not recognized conserved sequence features of this group of modifying C domains, including modifications of the highly conserved active site HHxxxDG motif for certain domains.

Our network analysis unveiled the *crz* BGC and closely related homologues thereof to be surprisingly widely distributed among bacteria from different phyla and allowed a comparative global analysis of the corallorazine-like GCF. In several cases, the presence of an additional MOx domain within the glycine-incorporating A domain indicates the production of oxidized or smaller, linear corallorazine derivatives. The widespread occurrence of BGCs for the assembly of corallorazine-like compounds suggests specialized bioactivities and ecological relevance of the encoded molecules, which may also be further investigated in view of their pharmaceutical potential. Finally, unexpected frequent co-occurrence of genes encoding a P450 monooxygenase and its corresponding FMN reductase along with the corallorazine NRPS genes in homologous BGCs give reason for further investigation of their potential impact in corallorazine biosynthesis.

## Experimental

### Biological material and cultivation

The myxobacterial strain *Corallococcus coralloides* B035 was isolated from a soil sample, collected from Belgium.^31^ For bacterial growth, 250 μL of a cryo culture of *C. coralloides* B035 was placed on VY/2 agar plates (5.0 g L^−1^ yeast extract, 1.36 g L^−1^ CaCl_2_·2H_2_O, 0.5 mg L^−1^ vitamin B12 and 15.0 g L^−1^ agar). For cultivation in liquid medium, slices of agar, containing fruiting bodies of the myxobacterium, were inoculated in MD-1 + glucose medium (3.0 g L^−1^ casitone, 0.7 g CaCl_2_·2H_2_O, 2.0 g L^−1^ MgSO_4_·7H_2_O, and 2.2 g L^−1^ glucose·H_2_O) and grown at 30 °C for 7 days under shaking conditions of 140 rpm. For long term storage at −80 °C, the liquid bacterial cultures were mixed with an equal volume of 1% casitone. Liquid cultures of all *E. coli* were grown in LB medium supplemented with the appropriate antibiotic. The *E. coli* strains were cultivated at 220 rpm and 37 °C. *E. coli* precultures were inoculated from cryo-cultures stored at 180 °C.

### Genomic DNA isolation

*C. coralloides* B035 was grown in liquid MD1 + glucose medium at 30 °C (140 rpm) for seven days. Gel electrophoresis materials were supplied from Neolab, Germany. All other materials and substances were supplied by Roth, Germany. Genomic DNA isolation was performed as described in.^8^

### Molecular cloning

Oligonucleotide primers were synthesized by Eurofins Genomics. Primers listed in Table S5 were used for amplification of whole *crz* genes or the truncated NRPS domain constructs of *crz1*. The resulting products of Q5-PCR (NEB) were cloned into the multiple cloning site of pET28a, using the respective endonucleases and T4 ligase (both NEB). The resulting constructs were isolated from *E. coli* alpha-select silver (Bioline), analyzed *via* restriction digest and verified by Sanger sequencing. The final plasmids were then transformed into *E. coli* BAP1 for overexpression of recombinant hexahistidine-tagged proteins.

### Heterologous protein expression and purification

Expression of NRPS constructs was performed in *E. coli* BAP1 to ensure *in vivo* phosphopantetheinylation of the T domains.^32^ A glycerol stock of the respective *E. coli* BAP1 culture was used to inoculate 5 mL of LB medium, supplemented with 50 ng mL^−1^ kanamycin for plasmid selection and incubated at 37 °C, 220 rpm overnight. This densely-grown preculture was used to inoculate 250 mL TB in a baffled flask, supplemented with kanamycin. After roughly 2 h incubation (37 °C, 220 rpm), OD_600_ reached 0.7 and the culture was chilled on ice. Protein expression was induced by addition of 0.4 mM isopropyl-β-d-thiogalactopyranoside (IPTG). The culture was then incubated for additional 16 h (16 °C, 200 rpm). For extraction of the proteins, cells were harvested *via* centrifugation (10 000 × *g*, 4 °C, 2 min). The cells were resuspended in 5 mL lysis buffer (50 mM NaH_2_PO_4_, 10 mM imidazole, 300 mM NaCl, pH 8) per g pellet. The cells were lysed *via* sonification and the lysate centrifuged (12 000 × *g*, 4 °C), until lysate was clear. The supernatant, containing the hexahistidine-tagged protein was incubated on ice with 1 mL of Ni-NTA-agarose suspension (Qiagen) for 1 h at 100 rpm. The suspension was finally filtered with a propylene column (Qiagen), washed with 4 mL wash buffer (50 mM NaH_2_PO_4_, 20 mM imidazole, 300 mM NaCl, pH 8) and eluted in 2.2 mL elution buffer (50 mM NaH_2_PO_4_, 300 mM imidazole, 300 mM NaCl, pH 8). The buffer system of the elution fraction was exchanged with PD-10 columns (Cytiva), following the manufacturers protocol. The desired concentration as set by centrifugation with Amicon membrane filter (Merck KGaA) with a MWCO of 30 kDa or 10 kDa.

### γ^18^O_4_ATP-exchange assay

2 μL of the A domain containing NRPS (5 μM in 20 mM tris pH 7.5, 5% glycerol) was mixed with 2 μL solution 1 (3 mM amino acid, 15 mM pyrophosphate, 20 mM tris pH 7.5) and 2 μL solution 2 (3 mM γ^18^O_4_-ATP, 15 mM MgCl_2_, 20 mM tris pH 7.5) and incubated at 22 °C for 1 h. The reaction was quenched with 6 μL 9-aminoacridine in acetone (5 mg mL^−1^). Precipitated proteins were removed *via* centrifugation. For sample analysis, 1 μL of the sample were spotted on a carrier plate of ground steel and analyzed with a Bruker AutoFlex III MALDI-TOF-MS in negative mode. Absolute substrate conversion in [%] was calculated by dividing the peak area at *m*/*z* 506 through the combined peak areas at *m*/*z* 508, 510, 512, and 514, divided by 83.33 for the molar ratio of labelled against unlabeled pyrophosphate in the assay.^19^

### C_Starter_ domain assay

Crz1.1, with the domain architecture C_1_-A_1_-T_1_, was isolated from *E. coli* BAP1 and buffered to assay buffer (25 mM tris pH 7.5, 150 mM NaCl, 10 mM MgCl_2_) using PD-10 columns, following the manufacturers protocol. The assay was performed in a 500 μL one-pot-reaction: 12.5 μM Crz1.1 were incubated with 1 mM ATP, 1 mM l-Ser, and 0.5 mM octanoyl-CoA (CoALA) for 3 h at 22 °C. For the negative control, the enzyme was inactivated beforehand at 80 °C for 10 min. After incubation, proteins were precipitated with 10% trichloroacetic acid (final concentration) and incubated for 30 min on ice. Subsequently, the acid was removed by 3 washing steps with ddH_2_O. The precipitated proteins were then resuspended in 0.1 M KOH and incubated at 70 °C for 20 min to cleave the thioester-T domain bonds upon alkaline hydrolysis. Samples were dehydrated *via* lyophilization and dissolved in a minimal volume of MeOH for LC–MS analysis using a Waters Alliance e2695 Separation Module, equipped with a RP-18 column (Waters XBridge BEH Shield RP18 Column; 3.5 μm, 2.1 × 100 mm). A binary solvent mixture of A: 90% acetonitrile + 10% ammonium acetate (5 mM) pH 7.4 and B: 10% acetonitrile + 90% ammonium acetate (5 mM) pH 7.4 was used, with a flow rate of 0.3 mL min^−1^, starting at minute 0–5 with isocratic elution of 10% B, a gradient 10% B → 100% at minute 5–25, and again isocratic conditions of 100% B at minute 25–30. Injection volume of MeOH sample was 5 μL. Signals were detected in negative ion mode for total ion current (TIC) and for selected ion recording (SIR) at *m*/*z* = 230.1 for octanoyl-serine.

### Bioinformatic analyses of condensation domains

In order to generate a set of representative C domain sequences from bacterial BGCs, mainly a reduced set of sequences from Wheadon and Townsend^22^ was used. This set includes E, Cyc, ^L^C_L_, C_Starter_, C_modAA_, dual E/C and ^D^C_L_ domains from different biosynthetic pathways deposited on MIBiG.^23^ To ensure a broad taxonomic coverage and to include additional known subtypes (hybridC, X and I) in the analysis, this set was extended with C domains from further bacterial biosynthesis pathways, which were also retrieved from clusters deposited on MIBiG or taken from literature sources. Selection of C_modAA_ sequences was based on the set used by Patteson *et al.*^12^ and extended by manual searches in the MiBIG repository. Extraction of domain sequences was done *via* InterPro^33^ using the general profile entry for C domains (IPR001242) with occasionally extending sequences on the sides, similarly to the procedure in.^22^ Multiple sequence alignments of C domain sequences were performed using MUSCLE v3.8.31^34^ algorithm implemented in Jalview v2.11.3.2^35^ with default settings. Resulting alignments were then manually trimmed at both ends in order to remove overhangs and poorly aligned regions. Alignments were displayed as sequence logos using WebLogo 3.^36^ For better visualization and comparability, alignment positions with gaps of more than 90% were removed for generation of logos, with the exception of affected positions in the sequences of Crz1 C_2_ and AmbE C_2_ from the C_modAA_ alignment. C domain core motifs were identified manually according to He *et al.*^37^ and Rausch *et al.*^38^ Phylogenetic reconstructions were then performed using raxmlGUI v2.0.10,^39^ an application that provides a graphical interface for the phylogeny tool RAxML v8.0.^40^ First, applying ModelTest-NG,^41^ which is implemented in raxmlGUI, the best substitution matrix for each alignment was determined. For both the set of different subtypes of the C domain superfamily, as well as the set comprising only C_modAA_ domains, the LG model^42^ was the best fit, with the decorations +I +G4 +F being determined for the former set and +G4 +F for the latter. With these settings, a maximum likelihood tree with 100 bootstrap replicates was created and visualised with ITOL v.6.^24^

### Structural model of Crz1-C_2_

The structure prediction for Crz1-C_2_ was retrieved with AlphaFold v2.3.2 Colab^43^ using default settings. For this purpose, Crz1-C_2_ was specified ranging from position 1076 to 1516 within the protein sequence of Crz1 (QAT85340.1). Visualization was done using UCSF ChimeraX v1.7.1.^43^

### Bioinformatic analyses of corallorazine BGC and homologues

The representatively chosen amino acid sequences of the proteins Crz1 (QAT85340.1), Crz2 (QAT85339.1) and Crz4 (QAT85337.1), which were considered characteristic for the corallorazine BGC, were each subjected to a global alignment with sequences from the National Center for Biotechnology Information (NCBI) databank, using protein–protein BLAST^44^ at standard parameters with filter setting from 41–100% identity. Nucleotide regions, on which the matched protein's coding regions were localized, were screened for BGCs using antiSMASH 7.1.0^9^ in relaxed mode. Corresponding annotated BGC containing regions where collected and subjected to a BiG-SCAPE analysis^26^ using mixed mode at a 0.7 cutoff level. The resulting network was visualized with Cytoscape 3.5.1. Pre-sorted gene clusters were revisited and manually categorized, based on the presence of genes homologous to *crz1–8*. Some examples of gene clusters along neighboring regions were visualized using Gene Graphics^45^ and genes were colorized according to their predicted product, if information was available.

### Determination of minimum inhibitory concentrations (MICs)

Corallorazine A was isolated from *Corallococcus coralloides* B035 as previously described.^7^ MICs were determined by broth micro-dilution according to CLSI guidelines. Test medium was cation-adjusted Mueller–Hinton broth (MHB) and cell concentrations were adjusted to approximately 5 × 10^5^ cells per ml. After 20 h of incubation at 37 °C, the MIC was defined as the lowest concentration of antibiotic that inhibited visible growth.

### *Bacillus subtilis* β-galactosidase reporter assays

*B. subtilis* β-galactosidase reporter assays were performed as previously described.^46^ In short, the reporter strains *B. subtilis* 168 pAC6-P_*ypuA*_ (cell wall), pAC6-P_*yorB*_ (DNA), pAC6-P_*yvgS*_ (RNA) and pAC6-P_*yheI*_ (protein)were grown in MHB supplemented with 5 μg mL^−1^ chloramphenicol at 30 °C to an OD_600_ of 0.5. MHA plates containing 5 μg mL^−1^ chloramphenicol with 150 μg mL^−1^ (cell wall reporter), 250 μg mL^−1^ (DNA reporter), and 500 μg mL^−1^ (RNA and protein reporter) X-gal, respectively, and 1 × 10^7^ CFU mL^−1^ of the respective strain were poured at 55 °C. After cooling down, 5 μg corallorazine A as well as positive and negative control antibiotics (6 μg vancomycin and 3 μg clindamycin for cell wall, 0.3 μg ciprofloxacin and 3 μg clindamycin for DNA, 6 μg rifampin and 3 μg clindamycin for RNA, 3 μg clindamycin and 6 μg vancomycin for protein reporter) were spotted and allowed to dry before incubating overnight at 30 °C.

### Susceptibility profiling of *S. aureus* strains, depleted for proteins involved in transcription and translation mediated by antisense RNA expression

*S. aureus* RN4220 strains, in which specific antisense RNAs were conditionally expressed to reduce expression levels of proteins involved in transcription and translation, were tested for susceptibility towards corallorazine A as reported previously.^47^ First, the xylose concentration which inhibits the visible growth of the respective antisense strains was determined as described above (determination of MICs). Overnight cultures grown in LB containing 34 μg mL^−1^ chloramphenicol were diluted to an OD_600_ of 0.01 in the LB medium supplemented with 0×, 0.25×, 0.5× and 1× MIC of xylose, respectively. Cell suspensions were transferred into a transparent 96 chimney well plate (Greiner) containing serially diluted corallorazine A or ampicillin as control. Growth was monitored for 16 h at 37 °C using a Tecan Spark 10 M microplate reader. The increase in susceptibility was calculated as previously described.^48^

### *In vitro* transcription using *E. coli* RNA polymerase

Unless otherwise stated, transcription was assessed using 0.5 units *E. coli* RNA polymerase holoenzyme (New England Biolabs), 0.5 μg of ΦX174 RF DNA, and corallorazine A in indicated amounts in 1× reaction buffer (40 mM Tris–HCl, 150 mM KCl, 10 mM MgCl_2_, 1 mM dithiothreitol, 0.01% Triton X-100, 0.5 mM of each NTP) in a total volume of 25 μL. The DNA was transcribed at 37 °C for 2 h. After the addition of 25 μL 2x RNA loading dye (ThermoFisher Scientific), the reaction was incubated at 70 °C for 10 min and immediately cooled down on ice for another 3 min. The samples were loaded onto a 1% agarose gel supplemented with 1× GelRed® (Biotium) and run at 100 V. The gel was imaged using the Molecular Imager® Gel Doc™ XR+ imaging system with the Image Lab Software Version (BioRad Laboratories).

## Conflicts of interest

There are no conflicts to declare.

## Supplementary Material

CB-005-D4CB00157E-s001

## Data Availability

The data supporting this article (tables of biosynthetic gene clusters and NRPS domains used for the bioinformatic analyses) have been included as part of the ESI.†
