# Supplementary material for: Biosynthesis of the corallorazines, a widespread class of antibiotic cyclic lipodipeptides
Source: RSC Chem Biol. 2024 Aug 16;5(10):970–80. doi: 10.1039/d4cb00157e (PMC11342130; doi:10.1039/d4cb00157e)
Supplement: CB-005-D4CB00157E-s001 [file CB-005-D4CB00157E-s001.pdf]

Supplementary Information (SI) for RSC Chemical Biology.
This journal is © The Royal Society of Chemistry 2024

Supplementary Information for

Biosynthesis of the corallorazines, a wide spread class of antibiotic cyclic lipodi-peptides

Contents

Supplementary Figures and Tables 2

Table S1: Overview and deduced functions of open reading frames encoding the corallorazine BGC *crz1*- *crz8* (grey box) and adjacent genes. 2

Figure S1: Proposed scheme of corallorazine biosynthesis. 3

Figure S2: SDS-PAGE of purified Crz1.1, Crz1.2 & Crz2. SDS-PAGE of protein fractions from expression of *E. coli* BAP1 expression strains holding respective expression plasmids. 4

Figure S3: Core motifs in Crz 1/2 AGly. 5

Figure S4: Unrooted maximum-likelihood bootstrap phylogeny trees of 275 sequences from the C domain superfamily. 6

Table S2: C domains from bacterial NRPS BGCs that cluster within the C_{modAA} clade. 7

Figure S5: Sequence alignment of 50 C_{modAA} domains. 8

Figure S7: Sequence logo of selected conserved motif sections for C domains from different subclades. 10

Figure S8: Structural model of Crz1-C_2 seen from donor side. 11

Figure S9: Structural model of Crz1-C_2 with highlighted conserved residues. 11

Table S3: Presence of crz homologues in BGCs from the BiG-SCAPE network analysis. 12

Figure S10: Cytoscape network of homologous corallorazine BGCs. 15

Figure S11: Examples of characteristic corallorazine-like BGCs from clade I-III. 16

Table S4: antiSMASH regions of BGCs with corallorazine-like genes 17

Table S5: Primers used for cloning of crz genes. 20

Supplementary References 21

Supplementary Figures and Tables

Table S1: Overview and deduced functions of open reading frames encoding the corallorazine BGC *crz1*- *crz8* (grey box) and adjacent genes.

Gene	Size (kb)	Highest homology (protein level)	Putative function	Identity of aligned amino acids	GenBank accession number
orf1	981	FHA domain-containing protein [*Corallococcus coralloides*]	signal transduction/regulation??	315/322 (98%)	WP_014396743.1
orf2	1350	serine protease [*Corallococcus coralloides*]	S1C family peptidase	438/449 (98%)	WP_014396742.1
orf3	669	hypothetical protein [*Corallococcus coralloides*]	hypothetical protein	218/221 (99%)	WP_014396741.1
crz1	7,131	non-ribosomal peptide synthetase [*Streptomyces hokutonensis*]	NRPS	1345/2381 (56%)	WP_019071574.1
crz2	1,716	NAD-dependent epimerase/dehydratase family protein [*Methylobacter tundripaludum*]	PCP reduction domain	368/566 (65%)	WP_104427995.1
crz3	1,815	fatty acyl-AMP ligase [*Streptomyces sp.*]	AMP-ligase	349/570 (61%)	WP_059249057.1
crz4	1,725	hypothetical protein [*Methylobacter tundripaludum*]	Acyl-CoA dehydrogenase	318/581 (55%)	WP_104427994.1
crz5	1,731	acyl-CoA dehydrogenase [*Methylobacter tundripaludum*]	Acyl-CoA dehydrogenase	310/577 (54%)	WP_104427993.1
crz6	261	polyketide synthase *[Methylobacter tundripaludum]*	acyl carrier protein	42/75 (56%)	WP_104427992.1
crz7	1,227	cytochrome P450 [*Methylobacter tundripaludum*]	cytochrome P450	265/401 (66%)	WP_104427991.1
crz8	579	FMN reductase [*Acidobacteria bacterium*]	NAD(P)H-dependent oxidoreductase	95/175 (54%)	PYT83693.1
orf12	31,506	non-ribosomal peptide synthetase [*Corallococcus coralloides*]	NRPS/long-chain fatty acid CoA ligase	10289/10504 (98%)	WP_014396740.1
orf13	1602	MBL fold metallo-hydrolase [*Corallococcus coralloides*]	putative antibiotic biosynthesis protein (ß-lactamase)	523/533 (98%)	WP_014396739.1
orf14	1026	CPBP family intramembrane metalloprotease [*Corallococcus coralloides*]	resistance??	326/341 (96%)	WP_014396738.1

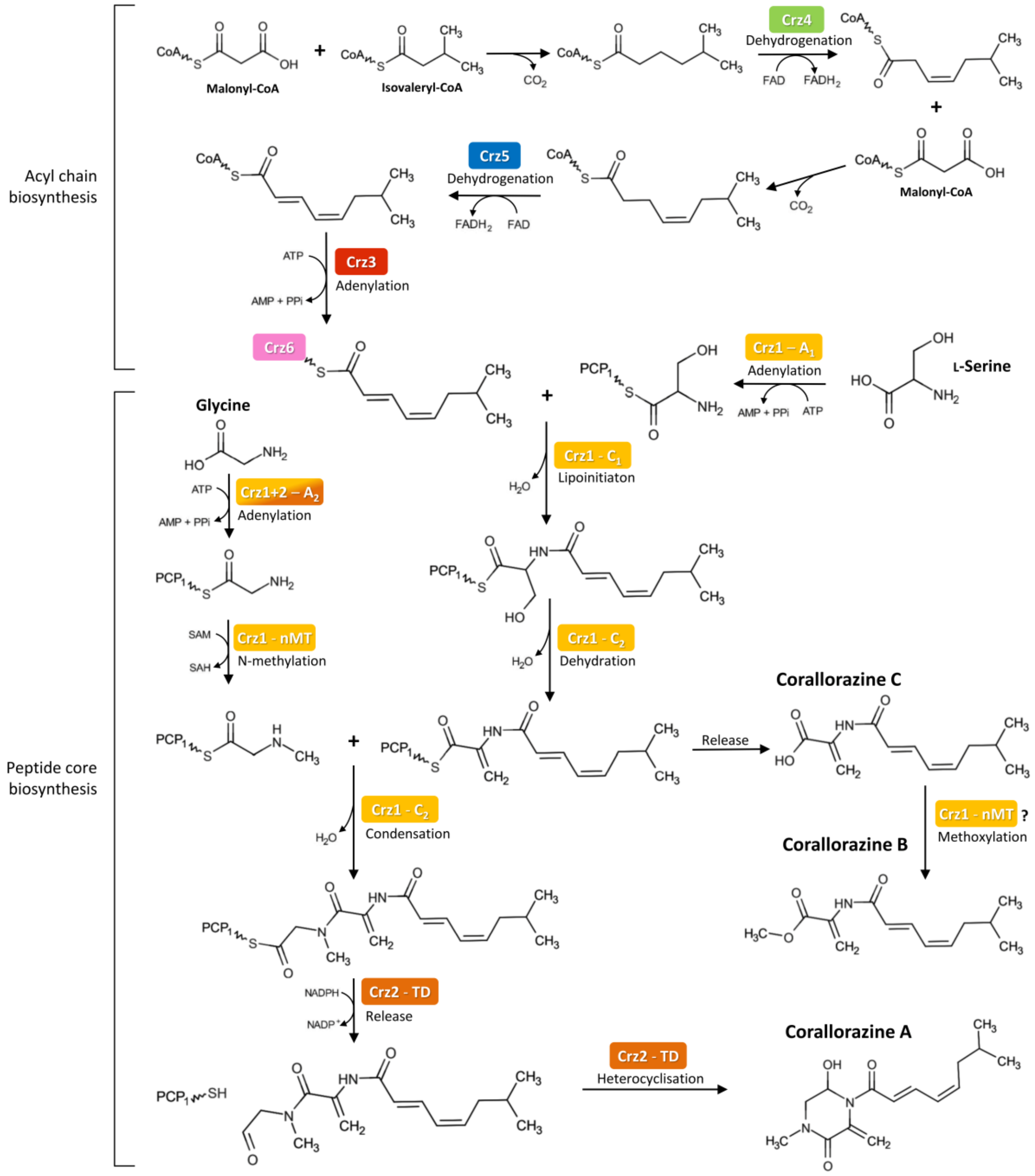

Figure S1: Proposed scheme of corallorazine biosynthesis. For each reaction step, the respective catalyzing enzymatic domain of proteins Crz1-6, indicated in different colors, is shown.

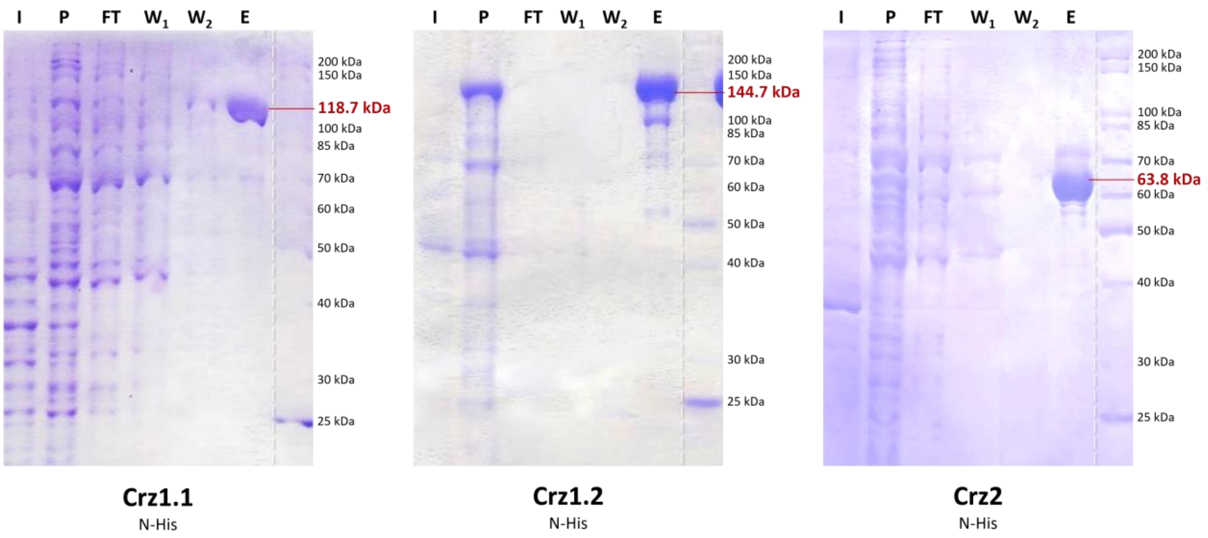

Figure S2: SDS-PAGE of purified Crz1.1, Crz1.2 & Crz2. SDS-PAGE gels of protein fractions from expressions of *E. coli* BAP1 cultures containing respective expression plasmids. I = pellet from induced culture; P = insoluble cell pellet of lysed culture; FT = non-binding flow-through; W_1 = wash buffer I, W_2 = wash buffer II; E = elution fraction. Sizes of expected bands are shown in red. NEB Unstained Protein Standard Broad Range (10-200 kDa) ladder was used as size reference. Proteins were separated at 140 V on 12% SDS gels and stained with Coomassie Blue.

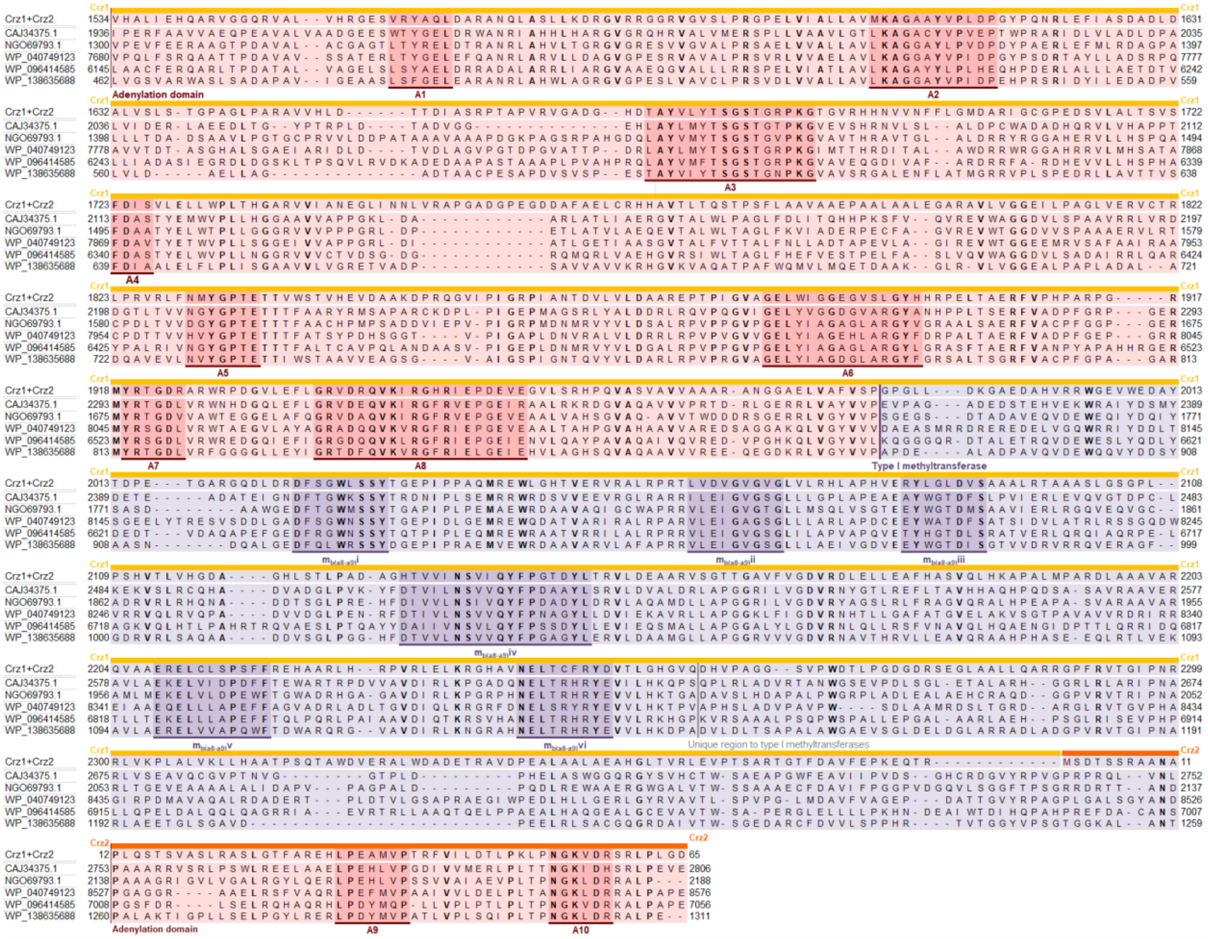

Figure S3: Core motifs in Crz 1/2 AGly. Alignment of A_{Gly} located on Crz1 (aa 1534 - 2376) plus Crz2 (aa 1 - 65) with further type I methyltransferase interrupted A domains according to Lundy et al. [1] Core motifs A1-10 [2,3] of A domains (red) as well as consensus motifs $m_{b(a8\text{-}a9)}$i-vi and the unique region of type I MTs (purple) are indicated below. Amino acids residing on Crz1 are indicated by the upper yellow bar and those on Crz2 by the upper orange bar.

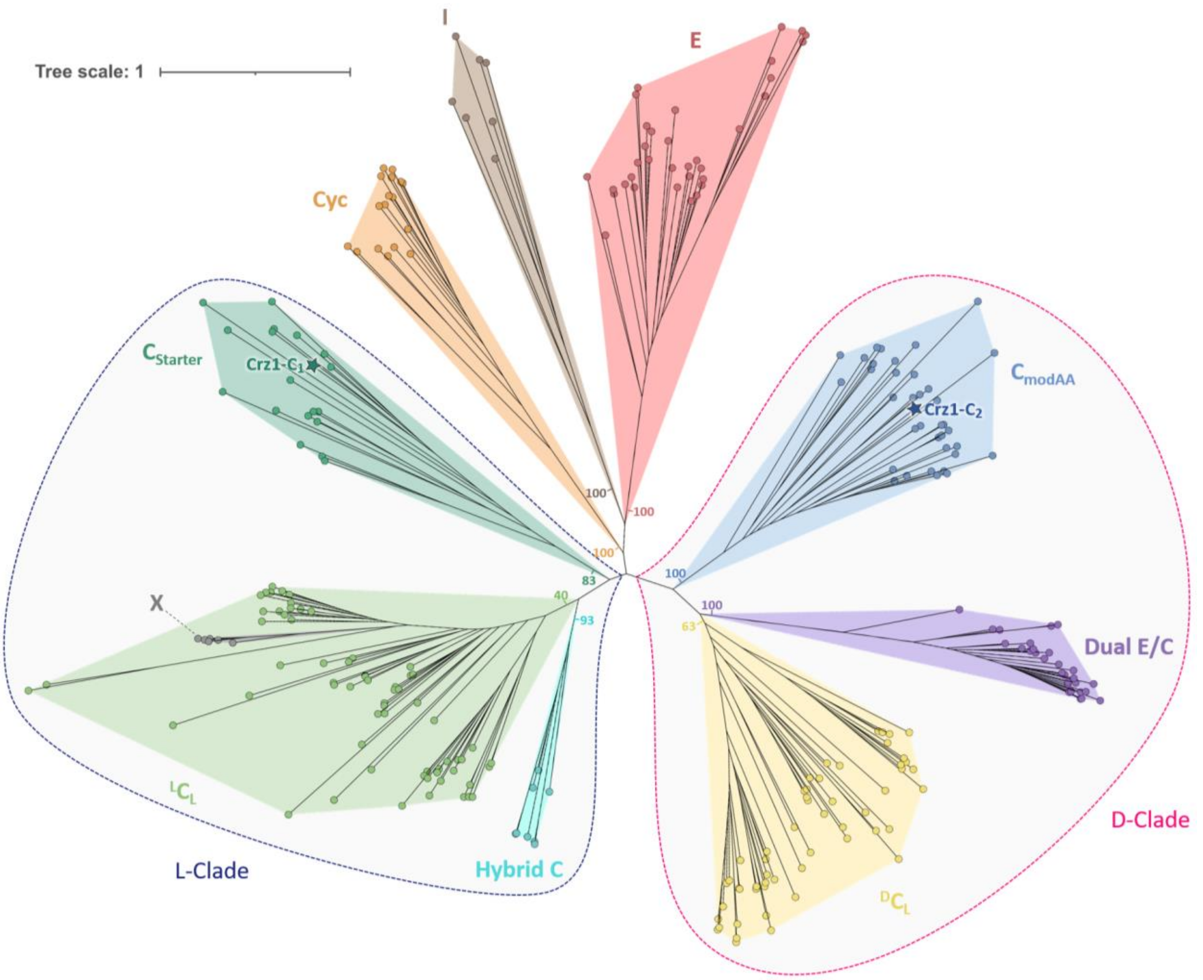

Figure S4: Unrooted maximum-likelihood bootstrap phylogeny trees of 275 sequences from the C domain superfamily. Clades representing different functional subtypes are highlighted in different colours and provided with bootstrap values. Following subtypes are included in the tree: Heterocyclization (Cyc, orange), Interface (I, brown), Epimerization (E, red), dehydrating/amino acid-modifying (C_{modAA}, blue), Dual epimerization/condensation (Dual E/C, purple), $^{D}C_{L}$ (yellow), Hybrid PKS/NRPS metabolite associated (Hybrid C, cyan), $^{L}C_{L}$ (light green), inactive & cytochrome P450 recruiting (X, grey), and Starter ($C_{Starter}$, dark green). Both C domains from the corallorazine biosynthetic pathway are indicated by asterisks. The L- and D-clades are circled in dark blue and pink. Visualisation was done with iTol v6 [4].

Table S2: C domains from bacterial NRPS BGCs that cluster within the C_{modAA} clade. Listed are C_{modAA} domains sorted according to the final modification of the amino acid they act on, as classified by Patteson et al. [5], being either dehydration only (DH, blue), L-2-amino-4-methoxy-*trans*-3-butenoic acid formation (AMB, purple), pyrrolizidine alkaloid formation (PA, green), pyrimidine formation (PY, orange), conjugate addition on side chain (CA, red) or conjugate addition on side chain instead of amide formation (CA*, yellow). In addition to domains already reported by Patteson et al., the list contains further potential C_{modAA} domains identified here by analyses of MIBiG 3.1 [6] and recent literature.

	C Domain	NCBI Protein Accession Nr.	Biosynthesis Pathway	MIBiG Pathway Accession Nr.	Producer organism	Reference (PMID)
DH	**AlbB-C_1**	KAA6212234.1	Albopeptide	-	*Streptomyces albofaciens* JCM 4342	33107670
	**AlbB-C_2**	KAA6212234.1	Albopeptide	-	*Streptomyces albofaciens* JCM 4342	33107670
	**Arg2-C_3**	QCE43602.1	Argyrin	BGC0001834	*Cystobacter* sp. SBCb004	30995838
	**BogB-C_1**	ATY37589.1	Bogorol	BGC0001532	*Brevibacillus laterosporus* DSM 25	27694801
	**CirA8-C_2**	BEV36892.1	Cirratiomycin	-	*Streptomyces cirratus* 248-Sq2	38456538
	**Crz1-C_2**	QAT85340.1	Corallorazine	-	*Corallococcus coralloides* B035	This study
	**DepE-C_2**	ABP57749.1	Romidepsin (FK228/FR901228)	BGC0000993	*Chromobacterium violaceum* 968	21183645
	**HasO-C_2**	AHZ20774.1	Hassallidin C	BGC0000369	*Anabaena* sp. Syke748	24742428
	**HasO-C_2**	CZT62784.1	Hassallidin E	BGC0001614	*Planktothrix serta* PCC 8927	28489343
	**HbcE-C_2**	MDK4218996.1	Herbicolin A	-	*Pantoea agglomerans* 9Rz4	36528875
	**HptC-C_2**	AJK45701.1	Haereoplantin	-	*Burkholderia plantarii* PG1	32808751
	**HptC-C_3**	AJK45701.1	Haereoplantin	-	*Burkholderia plantarii* PG1	32808751
	**JagA-C_2**	CCJ67645.1	Jagaricin	BGC0001127	*Janthinobacterium agaricidamnosum* DSM 9628	23161559
	**JagC- C_2**	CCJ67647.1	Jagaricin	BGC0001127	*Janthinobacterium agaricidamnosum* DSM 9628	23161559
	**LxaC-C_3**	KYC42747.1	Scytocyclamide A	BGC0002484	*Scytonema hofmannii* PCC 7110	33042096
	**LxaC1-C_3**	QSJ20135.1	Heinamide A1-3	BGC0002572	*Nostoc* sp. UHCC 0702	34085692
	**McyA-C_1**	AAF00960.1	Microcystin	BGC0001017	*Microcystis aeruginosa* PCC 7806	11033079
	**McyA-C_1**	QCQ67879.1	Microcystin	BGC0002297	*Phormidium* sp. LP904c	31067786
	**McyA-C_1**	CAD29797.1	Microcystin	BGC0001015	*Planktothrix agardhii* NIVA-CYA 126/8	12511503
	**McyA-C_1**	AAO62586.1	Microcystin	BGC0001016	*Anabaena* sp. 90	14766543
	**McyA-C_1**	AQH32484.1	Microcystin	BGC0001667	*Fischerella sp. CENA161*	29154789
	**MxvA-C_2**	WCB70648.1	Myxovalargin	-	*Corallococcus coralloides* 1071	36603206
	**MxvC-C_9**	WCB70646.1	Myxovalargin	-	*Corallococcus coralloides* 1071	36603206
	**MxvD-C_5**	WCB70647.1	Myxovalargin	-	*Corallococcus coralloides* 1071	36603206
	**NdaA-C_1**	ATP76243.1	Nodularin	BGC0001705	*Nostoc* sp. CENA543	29062311
	**NdaA-C_1**	AEO14743.1	Nodularin	BGC0000396	*Nostoc* sp. 73.1	22456448
	**Odl4-C_3**	CEK23364.1	Odilorhabdin NOSO-95 A-C	BGC0001716	*Xenorhabdus nematophila* AN6/1	29625040
	**PuwF-C_2**	AIW82283.1	Puwainaphycin	BGC0001125	*Cylindrospermum alatosporum* CCALA 988	25369527
	**PuwF-C_2**	AXN93614.1	Puwainaphycin	BGC0001953	*Anabaena* sp. UHCC-0399	30504214
	**PuwG-C_1**	AIW82284.1	Puwainaphycin	BGC0001125	*Cylindrospermum alatosporum* CCALA 988	25369527
	**PuwG-C_1**	AXN93615.1	Puwainaphycin	BGC0001953	*Anabaena* sp. UHCC-0399	30504214
	**ThiF-C_2**	UYH37519.1	Thiamyxins	-	*Myxococcaceae bacterium* MCy9487	36208117
	**VioC-C_2**	AWI62628.1	Vioprolide	BGC0001822	*Cystobacter* sp. Cb vi35	29694699
	**Zel5-C_2**	QXJ21809.1	Zelkovamycin B-E	BGC0002370	*Actinomadura graeca* 32-07	33216552
AMB	**AmbE- C_1**	AAG05690.1	Methoxyvinylglycine (AMB)	BGC0000287	*Pseudomonas aeruginosa* PAO1	29633497
PA	**AzeB-C_2**	AAG06715.1	Azabicyclene	BGC0002037	*Pseudomonas aeruginosa* PAO1	31247735
	**AzeB-C_2**	AEW95634.1	Azabicyclene	BGC0002697	*Streptomyces cattleya* NRRL 8057 = DSM 46488	35246663
	**BhmJ-C_2**	OKJ61999.1	Bohemamine A-C	BGC0002147	*Streptomyces* sp. CB02009	32463693
	**BraB-C_2**	CDF96614.1	Brabantamide	BGC0001149	*Pseudomonas* sp. SHC52	24436210
	**LgnD-C_2**	AIZ66879.1	Legonmycin	BGC0002666	*Streptomyces* sp. *MA37*	35013184
	**PxaA-C_2**	WP_051462298.1	Pyrrolizixenamide A	BGC0001873	*Xenorhabdus szentirmaii* DSM 16338	26465655
PY	**BlmX-C_2**	AAG02355.1	Bleomycin	BGC0000963	*Streptomyces verticillus* ATCC15003	11048953
	**TlmX-C_2**	ABL74936.1	Tallysomycin	BGC0001048	*Streptoalloteichus hindustanus* ATCC 31158	17216057
	**ZbmX-C_2**	ACG60782.1	Zorbamycin	BGC0001058	*Streptomyces pilosus*	19081934
CA	**FclJ-C_2**	PHM26613.1	Fabclavine	BGC0001130	*Xenorhabdus budapestensis* DSM 16342	24532262
	**TnaC-C_3**	AVI26390.1	Theonellamide	BGC0001800	*Candidatus Entotheonella serta*	29439203
	**Zmn17-C_2**	CCM44337.1	Zeamine	BGC0001056	*Serratia plymuthica* RVH1	23349809
CA*	**BlmVI-C_2**	AAG02359.1	Bleomycin	BGC0000963	*Streptomyces verticillus* ATCC15003	11048953
	**TlmVI-C_2**	ABL74940.1	Tallysomycin	BGC0001048	*Streptoalloteichus hindustanus* ATCC 31158	17216057
	**ZbmVI-C_2**	ACG60776.1	Zorbamycin	BGC0001058	*Streptomyces pilosus*	19081934

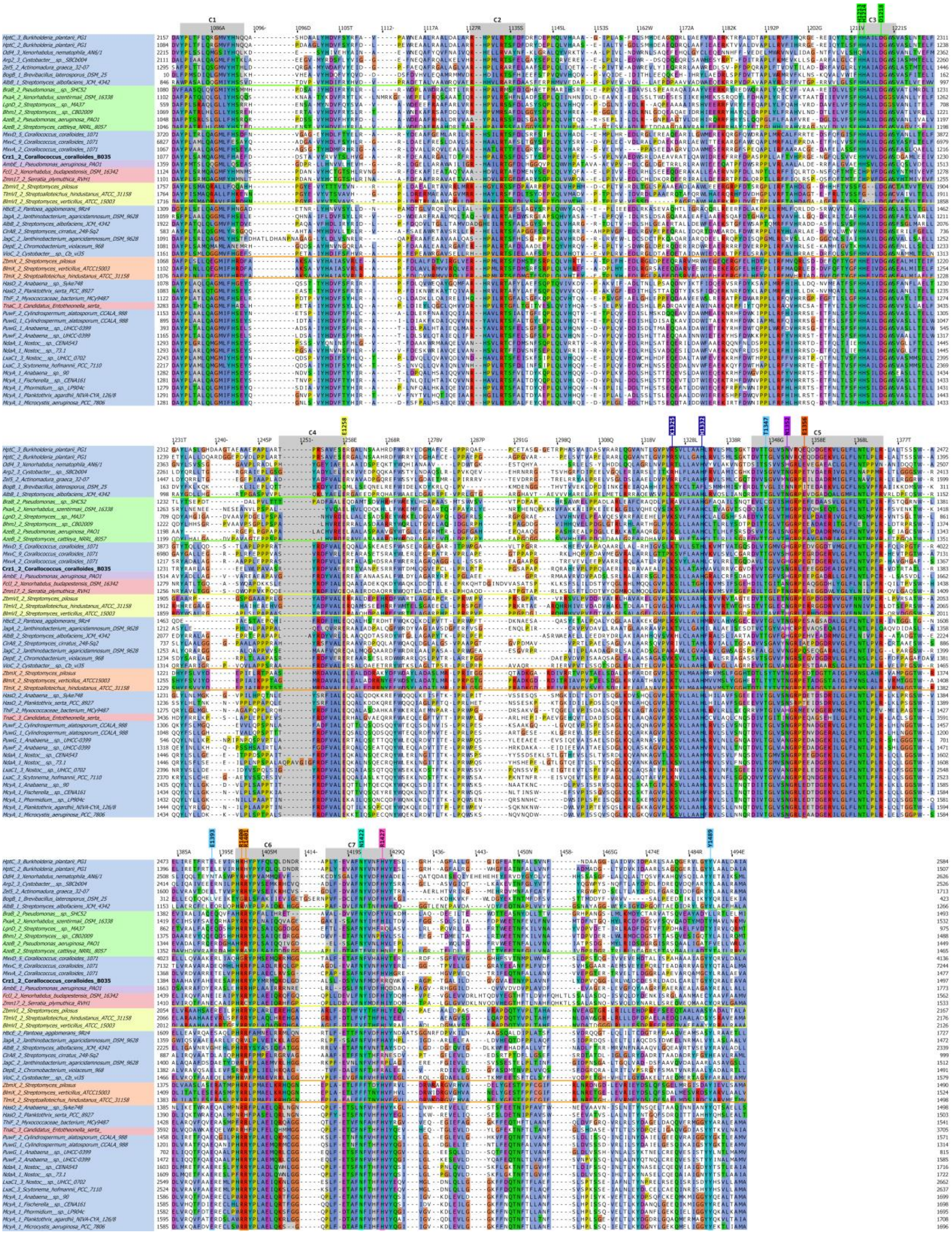

Figure S5: Sequence alignment of 50 C_{modAA} domains. Domain names are colored according to their predicted function in Figure 3. Position scale above the alignment corresponds to the numbering in Crz1. Sites that are potentially important and specific for C_{modAA} domains, and C domain core motifs, are labelled above the alignment in accordance with Figure S6. Alignment was performed and visualized using Jalview [7].

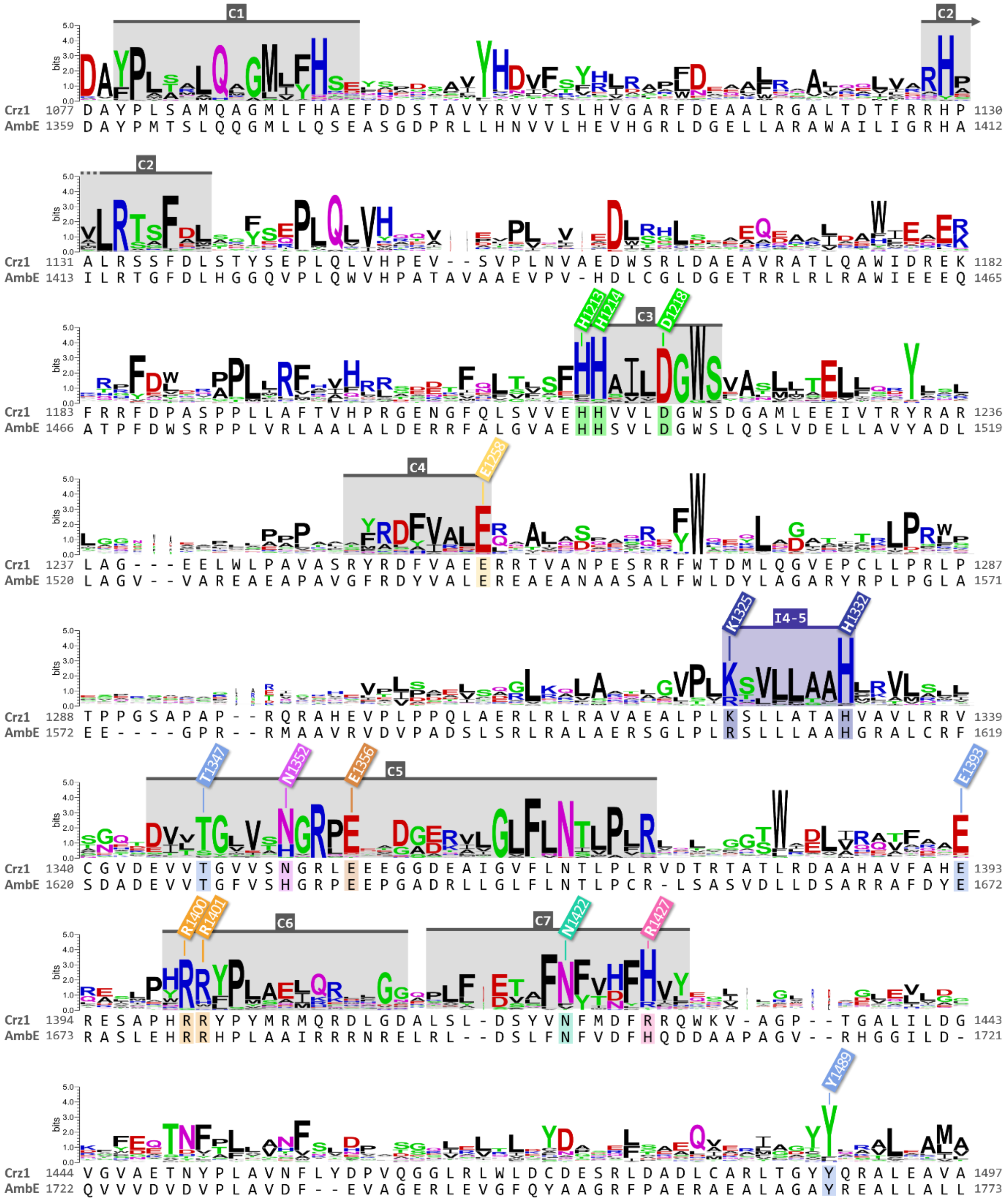

Figure S6: Sequence logo of C_{modAA} alignment of Crz-C_2 and AmbE-C_1. Selected residues are highlighted in different colors, the C_{modAA}-typical inter-motif "I4-5" is highlighted dark blue. Conserved C domain core motifs C1-7 (highlighted grey) were identified according to He et al. [8] and Rausch et al. [9]. Sequence logo was created using WebLogo 3 [10].

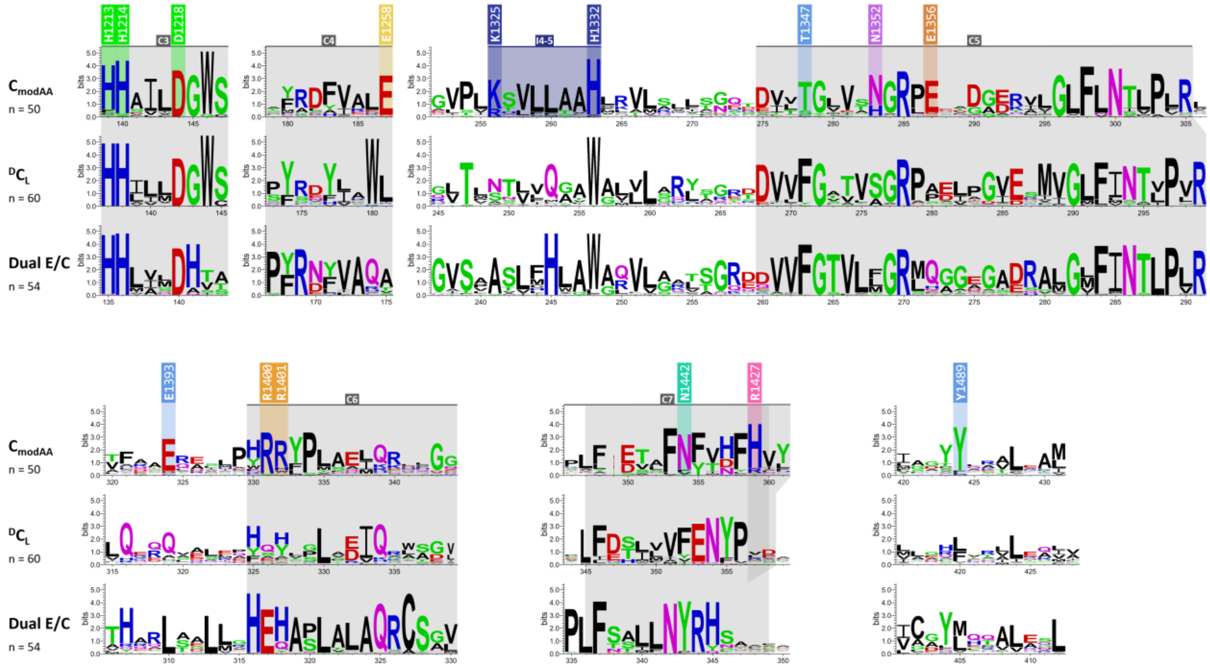

Figure S7: Sequence logo of selected conserved motif sections for C domains from different subclades. Selected residues are highlighted in different colors, the C_{modAA}-typical inter-motif "I4-5" is highlighted dark blue. Conserved C domain core motifs (highlighted grey) were identified according to He et al. [8] and Rausch et al. [9]. Sequence logos were created using WebLogo 3 [10].

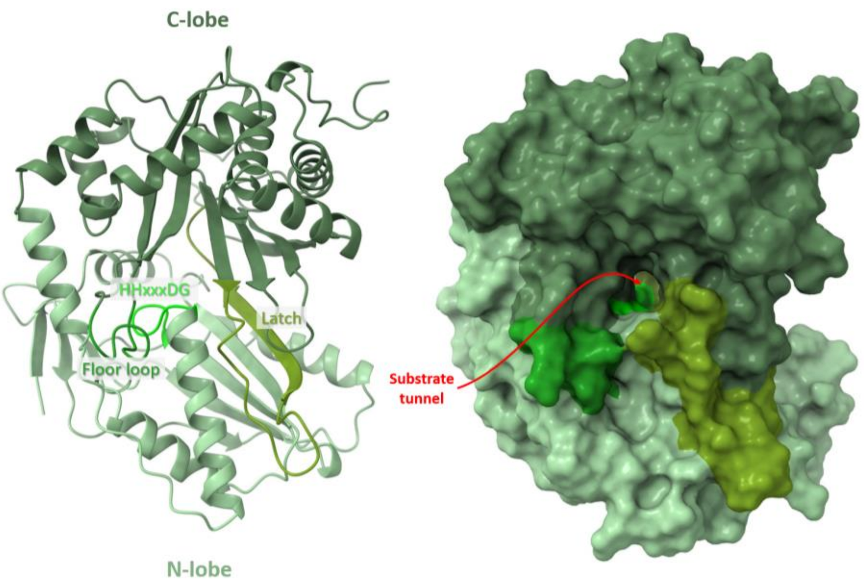

Figure S8: Structural model of Crz1-C_2 seen from donor side. Substrate tunnel, floor loop and latch are highlighted. Visualization was done using ChimeraX [11].

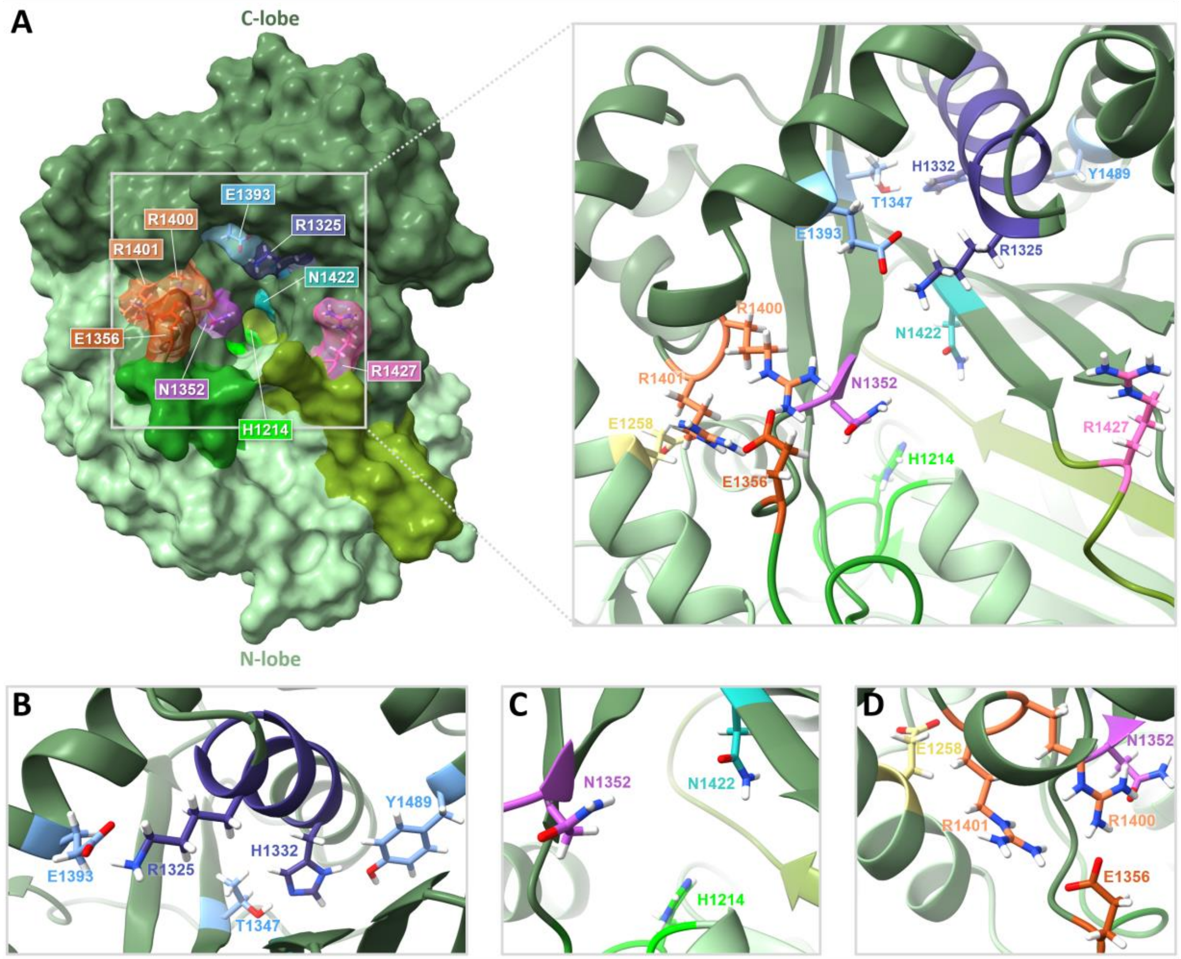

Figure S9: Structural model of Crz1-C_2 with highlighted conserved residues. Shown is a close-up view of the substrate tunnel seen from donor side (A) shown from different angles of the model (B-D). Visualization was done using ChimeraX [11].

Table S3: Presence of crz homologues in BGCs from the BiG-SCAPE network analysis. Colors of labels correspond to nodes in the network in Figure S10. NCBI accession numbers can be retrieved from Table S4.

**+**	**homologous gene is present**	**?**	**no prediction on presence of homologous genes**
++	duplicated homologous gene is present	f	genes form a fused gene
(+)	gene with analogous function is present more distantly	d	gene is divided
-	no homologous gene found	O	Crz1 A_{Gly} bears an integrated oxidase domain

Clade I – all corallorazine BGC genes		*crz*							
		1	*2*	*3*	*4*	*5*	*6*	*7*	*8*
A1	*Corallococcus coralloides*	+	+	+	+	+	+	+	+
A2	*Corallococcus exiguus*	+	+	+	+	+	+	+	+
A3	*Corallococcus llansteffanensis*	+	+	+	+	+	+	+	+
A4	*Corallococcus aberystwythensis*	+	+	+	+	+	+	+	+
A5	*Corallococcus praedator*	+	+	+	+	+	+	+	+
A6	*Corallococcus* sp. CA031C	+	+	+	+	+	+	+	+
A7	*Corallococcus* sp. CA047B	+	+	+	+	+	+	+	+
A8	*Corallococcus sicarius*	+	+	+	+	+	+	+	+
A9	*Myxococcaceae bacterium*	+	+	+	+	+	+	+	+
A10	*Cellulomonas phragmiteti*	+	+	+	+	+	+	+	+
A11	*Methylobacter tundripaludum*	+	$+^{f}$	$+^{f}$	+	+	+	+	+
A12	*Streptomyces tricolor*	+	+	+	+	+	+	++	+
A13	*Streptomyces hokutonensis*	+	+	+	+	+	+	++	+
A14	*Streptomyces echinatus*	+	+	+	+	+	+	++	+
A15	*Streptomyces* sp. CNQ-509	+	+	+	+	+	+	++	+
A16	*Streptomyces* sp. CNZ287	+	+	+	+	+	+	++	+
A17	*Streptomyces mexicanus*	+	+	+	+	+	+	++	+
A18	*Streptomyces* sp. FBKL.4005	+	+	+	+	+	+	++	+
A19	*Streptomyces reticuli*	+	+	+	+	+	+	++	+
A20	*Streptomyces* sp. EAS-AB2608	+	+	+	+	+	+	++	+
A21	*Streptomyces* sp. PBH53	+	+	+	+	+	+	++	+
A22	*Streptomyces tsukubaensis*	+	+	+	+	+	+	++	+
A23	*Streptomyces tsukubaensis*	+	+	+	+	+	+	++	+
A24	*Streptomyces* sp. NA04227	+	+	+	+	+	+	++	+
A25	*Streptomyces* sp. TS71-3	+	+	+	+	+	+	++	+
A26	*Pseudomonas mandelii*	$+^{O}$	+	+	+	+	+	+	+
A27	*Pseudomonas gingeri*	$+^{O}$	+	+	+	+	+	+	+
A28	*Pseudomonas chlororaphis*	$+^{O}$	+	+	+	+	+	+	+
A29	*Deinococcus cellulosilyticus*	$+^{O}$	+	+	+	+	+	+	+
A30	*Serratia plymuthica*	$+^{O}$	+	+	+	+	+	+	+
A31	*Serratia plymuthica*	$+^{O}$	+	+	+	+	+	+	+
A32	*Serratia plymuthica*	$+^{O}$	+	+	+	+	+	+	+
A33	*Serratia plymuthica*	$+^{O}$	+	+	+	+	+	+	+
A34	*Serratia sp.* PAMC26656	$+^{O}$	+	+	+	+	+	+	+
A35	*Serratia plymuthica*	$+^{O}$	+	+	+	+	+	+	+
A36	*Serratia plymuthica*	$+^{O}$	+	+	+	+	+	+	+
A37	*Serratia plymuthica*	$+^{O}$	+	+	+	+	+	+	+
A38	*Serratia plymuthica*	$+^{O}$	+	+	+	+	+	+	+
A39	*Serratia plymuthica*	$+^{O}$	+	+	+	+	+	+	+
A40	*Serratia marcescens*	$+^{O}$	+	+	+	+	+	+	+
A41	*Serratia marcescens*	$+^{O}$	+	+	+	+	+	+	+
A42	*Serratia marcescens*	$+^{O}$	+	+	+	+	+	+	+
A43	*Klebsiella oxytoca*	$+^{O}$	+	+	+	+	+	+	+
A44	*Serratia rubidaea*	$+^{O}$	+	+	+	+	+	+	+
A45	*Serratia rubidaea*	$+^{Od}$	+	+	$+^{d}$	$+^{d}$	+	+	+

Clade II –corallorazine NRPS-genes		crz							
		1	2	3	4	5	6	7	8
B1	*Streptomyces buecherae*	$+^{O}$	+	-	-	-	-	+	-
B2	*Streptomyces buecherae*	$+^{O}$	+	-	-	-	-	+	-
B3	*Streptomyces buecherae*	$+^{O}$	$+^{f}$	-	-	-	-	$+^{f}$	-
B4	*Streptomyces buecherae*	$+^{O}$	$+^{f}$	-	-	-	-	$+^{f}$	-
B5	*Streptomyces* sp. CB02980	$+^{O}$	+	-	-	-	-	+	-
B6	*Streptomyces ochraceiscleroticus*	$+^{O}$	+	-	-	-	-	+	++
B7	*Streptomyces roseicoloratus*	$+^{O}$	+	-	-	-	-	+	++
B8	*Streptomyces nashvillensis*	$+^{O}$	+	-	-	-	-	+	++
B9	*Streptomyces tanashiensis*	$+^{O}$	+	-	-	-	-	+	++
B10	*Streptomyces albus*	$+^{O}$	$+^{f}$	-	-	-	-	$+^{f}$	+
B11	*Streptomyces albus*	$+^{O}$	$+^{f}$	-	-	-	-	$+^{f}$	+
B12	*Streptomyces albus*	$+^{O}$	$+^{f}$	-	-	-	-	$+^{f}$	+
B13	*Actinokineospora baliensis*	$+^{O}$	+	(+)	-	-	-	+	+
B14	*Kitasatospora indigofera*	$+^{O}$	+	-	-	-	-	+	++
B15	*Kitasatospora cheerisanensis*	$+^{Odf}$	$+^{f}$	-	-	-	-	+	?
B16	*Kutzneria buriramensis*	-	+	+	+	+	+	(+)	-
B17	*Rhodococcus* sp. 852002-51564	$+^{O}$	+	-	+	+	+		
B18	*Saccharopolyspora* sp. 7K502	$+^{Od}$	+	+	?	?	?	?	?
B19	*Burkholderia glumae*	+	?	?	?	?	?	?	?
B20	*Nocardiopsis salina*	+	?	?	?	?	?	?	?

**Clade III** – non-corallorazine NRPS-genes		crz							
		1	2	3	4	5	6	7	8
C1	*Streptomyces* sp. CS149	-	-	+	+	+	+	-	-
C2	*Streptomyces olivaceus*	-	-	+	+	-	+	-	-
C3	*Streptomyces tendae*	-	-	+	+	-	+	-	-
C4	*Streptomyces* sp. M56	-	-	+	+	-	+	-	-
C5	*Streptomyces* sp. MH60	-	-	+	+	-	+	-	-
C6	*Streptomyces griseorubens*	-	-	+	+	-	+	-	-
C7	*Streptomyces* sp. VN1	-	-	+	+	-	+	-	-
C8	*Streptomyces parvulus*	-	-	+	+	-	+	-	-
C9	*Streptomyces* sp. RK74B	-	-	+	+	-	+	-	-
C10	*Streptomyces griseorubiginosus*	-	-	+	+	+	+	-	-
C11	*Streptomyces badius*	-	-	+	+	+	+	-	-
C12	*uncultured bacterium*	-	-	+	+	+	+	(+)	-
C13	*Streptomyces* sp. or43	-	-	+	+	+	-	-	-
C14	*Streptomyces* sp. TYQ1024	-	-	+	+	+	+	-	-
C15	*Streptomyces* sp. SID5643	-	-	-	+	+	+	(+)	-
C16	*Streptomyces ambofaciens*	-	-	+	+	+	+	-	-
C17	*Streptomyces ambofaciens*	-	-	+	+	+	+	-	-
C18	*Streptomyces luteoverticillatus*	-	-	+	+	+	+	-	-
C19	*Streptomyces janthinus*	-	-	+	+	+	+	-	-
C20	*Streptomyces collinus*	-	-	+	+	+	+	-	-
C21	*Streptomyces bellus*	-	-	+	+	+	+	-	-
C22	*Streptomyces coeruleorubidus*	-	-	+	+	+	+	-	-
C23	*Streptomyces violaceochromogenes*	-	-	+	+	+	+	-	-
C24	*Streptomyces achromogenes subsp. rubradiris*	-	-	+	+	+	+	-	-
C25	*Streptomyces* sp. WAC05858	-	-	+	+	+	+	-	-
C26	*Streptomyces* sp. AgN23	-	-	+	+	+	+	-	-
C27	*Streptomyces* sp. 11-1-2	-	-	+	+	+	+	-	-
C28	*Streptomyces mediolani*	-	-	+	+	+	+	-	-
C29	*Streptomyces* sp. SID5614	-	-	+	+	+	+	-	-
C30	*Actinomadura* sp. KC06	-	-	+	+	+	+	-	-
C31	*Actinomadura macra*	-	-	+	+	+	+	-	-
C32	*Plantactinospora mayteni*	-	-	+	+	+	+	-	-
C33	*Plantactinospora* sp. BB1	-	-	+	+	+	+	-	-
C34	*Plantactinospora* sp. BC1	-	-	+	+	+	+	-	-
C35	*Plantactinospora endophytica*	-	-	+	+	+	+	-	-

C36	*Plantactinospora* sp. CNZ320	-	-	+	+	+	+	-	-
C37	*Plantactinospora* sp. CNZ321	-	-	+	+	+	+	-	-
C38	*Plantactinospora soyae*	-	-	+	+	+	+	-	-
C39	*Streptomyces* sp. 67	-	-	+	+	+	+	-	-
C40	*Goodfellowiella* sp. AN110305	-	-	+	+	+	+	-	-
C41	*Lentzea kentuckyensis*	-	-	+	+	+	+	-	-
C42	*Streptomyces indicus*	-	-	+	+	+	+	(+)	(+)
C43	*Streptomyces* sp. CB03911	-	-	+	+	+	+	(+)	-
C44	*Streptomyces* sp. SID4936	-	-	+	+	+	+	(+)	-
C45	*Streptomyces actuosus*	-	-	+	+	+	+	(+)	-
C46	*Streptomyces* sp. CRXT-Y-14	-	-	+	+	+	+	(+)	-
C47	*Streptomyces griseosporeus*	-	-	+	+	+	+	(+)	-
C48	*Streptomyces* sp. CB09001	-	-	+	+	+	+	(+)	(+)
C49	*Kitasatospora* sp. MMS16-BH015	-	-	+	+	+	+	(+)	-
C50	*Streptomyces daqingensis*	-	-	+	+	+	+	(+)	-
C51	*Streptomyces* sp. 69	-	-	+	+	+	+	(+)	-
C52	*Streptomyces hiroshimensis*	-	-	+	+	+	+	(+)	(+)
C53	*Streptomyces flaveus*	-	-	+	+	+	+	(+)	(+)
C54	*Streptomyces* sp. 2231.1	-	-	+	+	+	+	(+)	(+)
C55	*Streptomyces* sp. CB01883	-	-	+	+	+	+	(+)	(+)
C56	*Streptomyces* sp. 840.1	-	-	+	+	+	+	(+)	(+)
C57	*Streptomyces* sp. 5112.2	-	-	+	+	+	+	(+)	(+)
C58	*Streptomyces* sp. PanSC9	-	-	+	+	+	+	(+)	(+)
C59	*Streptomyces* sp. SID9727	-	-	+	+	+	+	(+)	-
C60	*Streptomyces* sp. S3(2020)	-	-	+	+	+	+	-	-
C61	*Actinomadura logoneensis*	-	-	+	+	+	+	-	-
C62	*Streptomyces canus*	-	-	+	+	+	+	-	-
C63	*Streptomyces* sp. Root369	-	-	+	+	+	+	-	-
C64	*Streptomyces* sp. SID8350	-	-	+	+	+	+	-	-
C65	*Streptomyces* sp. SID8356	-	-	+	+	+	+	-	-
C66	*Streptomyces* sp. SID8352	-	-	+	+	+	+	-	-
C67	*Streptomyces* sp. 196(2019)	-	-	+	+	+	+	-	-
C68	*Streptomyces cavourensis*	-	-	+	+	+	+	-	-
C69	*Streptomyces* sp. KAI-26	-	-	+	+	+	+	-	-
C70	*Streptomyces* sp. CAI-24	-	-	+	+	+	+	-	-
C71	*Streptomyces* sp. SID8359	-	-	+	+	+	+	-	-
C72	*Streptomyces* sp. DvalAA-19	-	-	+	+	+	+	-	-
C73	*Streptomyces* sp. CFMR 7	-	-	+	+	+	+	-	-
C74	*Streptosporangium subroseum*	-	-	+	+	+	+	-	-
C75	*Streptomyces spongiae*	-	-	+	+	+	+	-	-
C76	*Microbispora* sp. RL4-1S	-	-	+	+	+	+	-	-
C77	*Phytohabitans rumicis*	-	-	+	+	+	+	-	-
C78	*Streptomyces pharetrae*	-	-	+	+	?	?	?	?
C79	*Streptomyces zaomyceticus*	-	-	-	+	-	-	-	-
C80	*Streptomyces* sp. NWU49	-	-	-	+	+	+	-	-
C81	*Salinispora tropica*	-	-	+	+	+	+	(+)	(+)

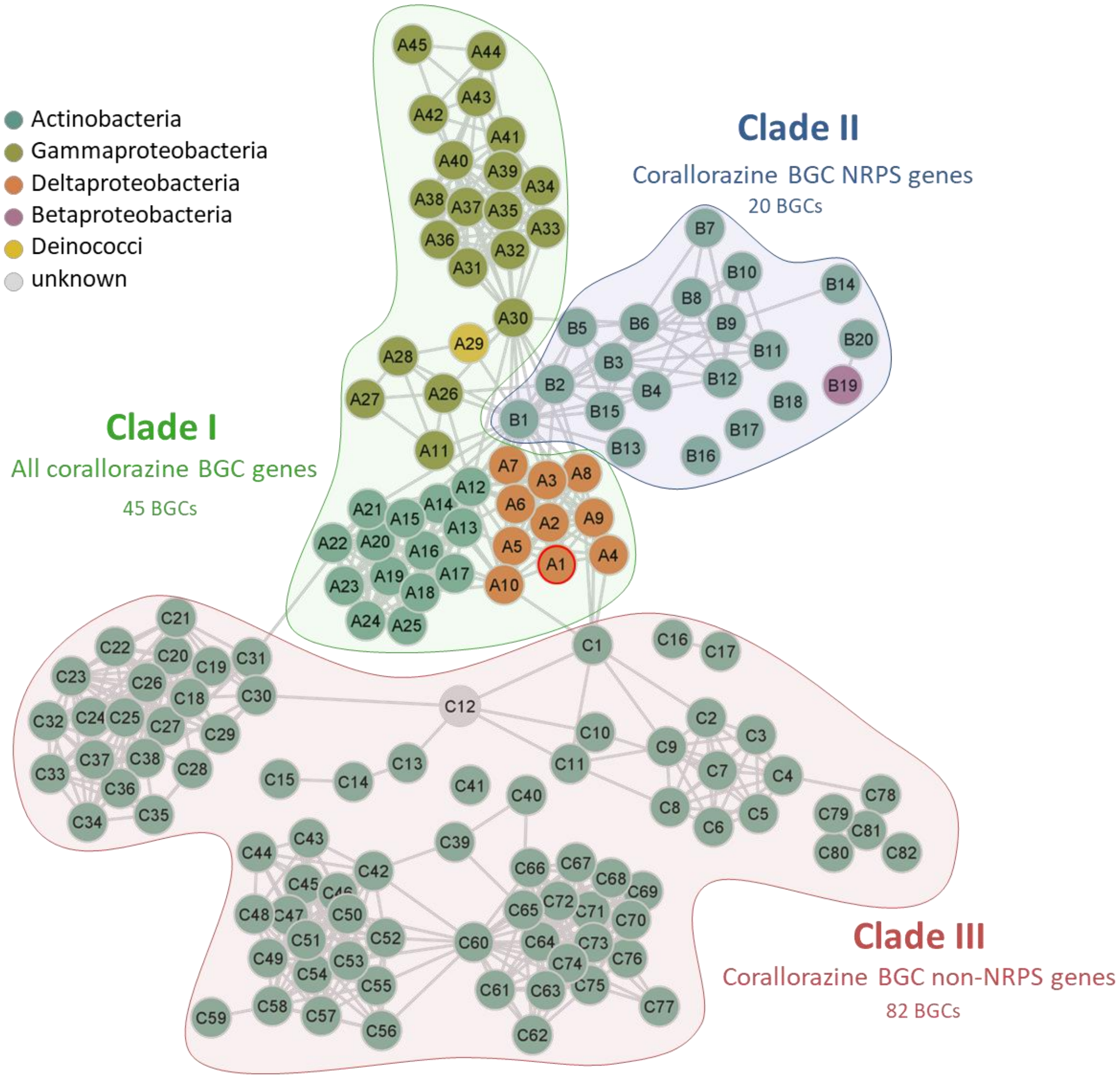

Figure S10: Cytoscape network of homologous corallorazine BGCs. Nodes correspond to BGCs containing at least one gene that is homologous to a gene in the corallorazine BGC (A1, red circle outline). Information on host strain and presence of *crz1-8* homologues is displayed in Table S3. Classification in clade I, II or III is indicated by colored areas. Examples for characteristic clusters from these clades are shown in Figure S11. Length of the connections between nodes correlates with the degree of relationship according to BiG-SCAPE analysis.

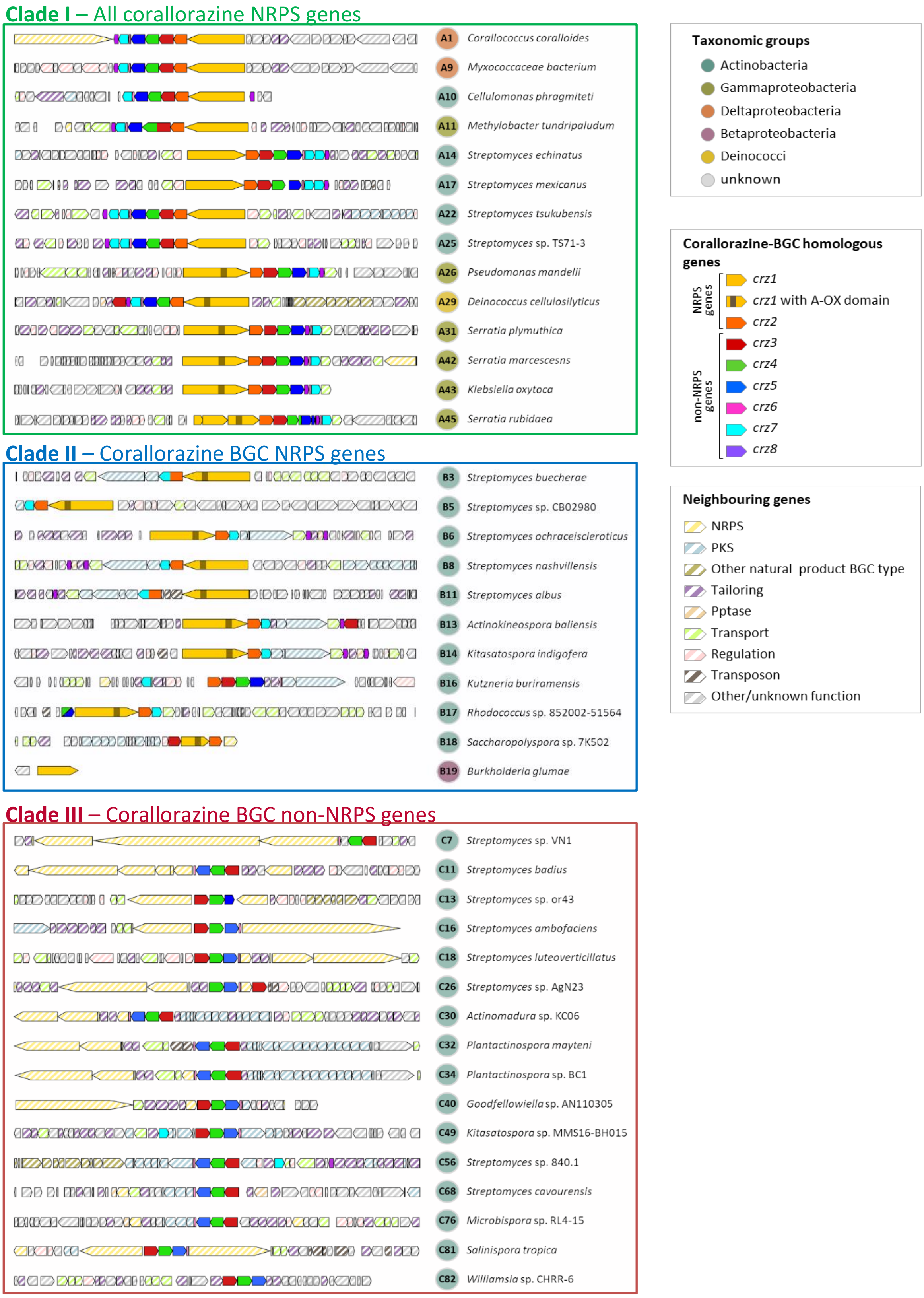

Figure S11: Examples of characteristic corallorazine-like BGCs from clade I-III. BGCs are listed with their respective node label from the network in Figure S10 and Table S3. Homologous genes to *crz1-8* from the original corallorazine BGC (BGC A1 in Clade I) are color-coded and genes in adjacent regions are labeled differently by functionality.

Table S4: antiSMASH regions of BGCs with corallorazine-like genes. The corresponding label of the node from the BiG-SCAPE analysis in and is given, along with the NCBI accession number of the nucleotide and the region detected by antiSMASH 7.0 [12], where the respective BGC is located. Regions marked with "*" are at the edge of a nucleotide contig and miss some part of information, which was placed on another contig.

Node label	NCBI Accession	antiSMASH Region	Species	Class
Clade I – All corallorazine BGC genes				
A1	CP034669.1	18	*Corallococcus coralloides*	Deltaproteobacteria
A2	JAFIMT010000001.1	14	*Corallococcus exiguus*	Deltaproteobacteria
A3	NZ_RAWB01000070	1*	*Corallococcus llansteffanensis*	Deltaproteobacteria
A4	RAWK01000026.1	1*	*Corallococcus aberystwythensis*	Deltaproteobacteria
A5	RAWI01000354.1	1*	*Corallococcus praedator*	Deltaproteobacteria
A6	RAWH01000308.1.1	1*	*Corallococcus* sp. CA031C	Deltaproteobacteria
A7	NZ_RAWD01000128	1*	*Corallococcus* sp. CA047B	Deltaproteobacteria
A8	NZ_RAWG01000099	1	*Corallococcus sicarius*	Deltaproteobacteria
A9	SECN01000053.1	1	*Myxococcaceae* bacterium	Deltaproteobacteria
A10	BONP01000021.1	1	*Cellulomonas phragmiteti*	Actinobacteria
A11	PTIZ01000002.1	1	*Methylobacter tundripaludum*	Gammaproteobacteria
A12	NZ_MUMF01000536	1*	*Streptomyces tricolor*	Actinobacteria
A13	NZ_BARG01000051	1	*Streptomyces hokutonensis*	Actinobacteria
A14	JACHJK010000001	3	*Streptomyces echinatus*	Actinobacteria
A15	NZ_CP011492.1	3	*Streptomyces* sp. CNQ-509	Actinobacteria
A16	NZ_VISV01000013	1	*Streptomyces* sp. CNZ287	Actinobacteria
A17	JACMHY010000005	3	*Streptomyces mexicanus*	Actinobacteria
A18	NZ_NPKF01000001	19	*Streptomyces* sp. FBKL.4005	Actinobacteria
A19	LN997842.1	15	*Streptomyces reticuli*	Actinobacteria
A20	AP024135.1	28	*Streptomyces* sp. EAS-AB2608	Actinobacteria
A21	NZ_CP011799.1	3	*Streptomyces* sp. PBH53	Actinobacteria
A22	NZ_CP045178.1	6	*Streptomyces tsukubaensis*	Actinobacteria
A23	MVFC01000001.	1	*Streptomyces tsukubaensis*	Actinobacteria
A24	NZ_CP054918.1	19	*Streptomyces* sp. NA04227	Actinobacteria
A25	BNEL01000003.1	3	*Streptomyces* sp. TS71-3	Actinobacteria
A26	NZ_RCZA01000006	1	*Pseudomonas mandelii*	Gammaproteobacteria
A27	JACAOS010000033	2	*Pseudomonas gingeri*	Gammaproteobacteria
A28	JAHTLD010000001	2	*Pseudomonas chlororaphis*	Gammaproteobacteria
A29	NZ_BJXB01000010	1	*Deinococcus cellulosilyticus*	Deinococci
A30	MQML01000149.1	1*	*Serratia plymuthica*	Gammaproteobacteria
A31	CP012096.1	6	*Serratia plymuthica*	Gammaproteobacteria
A32	CP006566.1	3	*Serratia plymuthica*	Gammaproteobacteria
A33	CP012097.1	3	*Serratia plymuthica*	Gammaproteobacteria
A34	JAEHKA010000016	1	*Serratia* sp. PAMC26656	Gammaproteobacteria
A35	JAAQRN010000002	2	*Serratia plymuthica*	Gammaproteobacteria
A36	CP002773.1	1	*Serratia plymuthica*	Gammaproteobacteria
A37	JADCNO010000024	1	*Serratia plymuthica*	Gammaproteobacteria
A38	JAEHSV010000004	2	*Serratia plymuthica*	Gammaproteobacteria
A39	CP007439.1	12	*Serratia plymuthica*	Gammaproteobacteria
A40	RCDL01000433.1	1	*Serratia marcescens*	Gammaproteobacteria
A41	JAFIZV010000023	1	*Serratia marcescens*	Gammaproteobacteria
A42	JAEHSX010000013	1	*Serratia marcescens*	Gammaproteobacteria
A43	FKZE01000007.1	1	*Klebsiella oxytoca*	Gammaproteobacteria
A44	CP065640.1	16	*Serratia rubidaea*	Gammaproteobacteria
A45	LR590463.1	11	*Serratia rubidaea*	Gammaproteobacteria
Clade II – Corallorazine BGC NRPS genes				
B1	JACOFO010000661	1*	*Streptomyces buecherae*	Actinobacteria
B2	JACOFP010000095	1*	*Streptomyces buecherae*	Actinobacteria
B3	NZ_CP054929.1	2	*Streptomyces buecherae*	Actinobacteria
B4	NZ_CP060404.1	30	*Streptomyces buecherae*	Actinobacteria
B5	JACSCH010000002	1*	*Streptomyces* sp. CB02980	Actinobacteria
B6	NZ_JOAX01000004	1	*Streptomyces ochraceiscleroticus*	Actinobacteria

B7	NZ_SDOY01000175	2	*Streptomyces roseicoloratus*	Actinobacteria
B8	BMUH01000002.1	1	*Streptomyces nashvillensis*	Actinobacteria
B9	BMRZ01000004.1	2	*Streptomyces tanashiensis*	Actinobacteria
B10	CP010519.1	1	*Streptomyces albus*	Actinobacteria
B11	CP033071.1	1	*Streptomyces albus*	Actinobacteria
B12	CP016825.1	1	*Streptomyces albus*	Actinobacteria
B13	JAFBCK010000001	26	*Actinokineospora baliensis*	Actinobacteria
B14	BNBO01000006.1	1	*Kitasatospora indigofera*	Actinobacteria
B15	JNBY01000152.1	1*	*Kitasatospora cheerisanensis*	Actinobacteria
B16	QUNO01000026.1	1	*Kutzneria buriramensis*	Actinobacteria
B17	LZMJ01000006.1	1	*Rhodococcus* sp. 852002-51564	Actinobacteria
B18	NZ_SMKW01000035	1*	*Saccharopolyspora* sp. 7K502	Actinobacteria
B19	JTDQ01000248.1	1*	*Burkholderia glumae*	Proteobacteria
B20	NZ_ANBF01000120	1*	*Nocardiopsis salina*	Actinobacteria
Clade III – Corallorazine BGC non-NRPS genes				
C1	PVZY01000056.1	1	Streptomyces sp. CS149	Actinobacteria
C2	NZ_JOFH01000027	1	Streptomyces olivaceus	Actinobacteria
C3	BMUY01000010.1	1*	Streptomyces tendae	Actinobacteria
C4	CP025018.1	8	Streptomyces sp. M56	Actinobacteria
C5	MULI01000006.1	1*	Streptomyces sp. MH60	Actinobacteria
C6	BMTJ01000005.1	5	Streptomyces griseorubens	Actinobacteria
C7	CP036534.1	20	*Streptomyces* sp. VN1	Actinobacteria
C8	NZ_QQBH01000019	1*	*Streptomyces* parvulus	Actinobacteria
C9	JAGPYE010000007	1*	*Streptomyces* sp. RK74B	Actinobacteria
C10	RJKZ01000001.1	16	*Streptomyces griseorubiginosus*	Actinobacteria
C11	NZ_BMSZ01000003	1	*Streptomyces badius*	Actinobacteria
C12	MK060022.1	1	uncultured bacterium	-
C13	NZ_RDBK01000025	1	*Streptomyces* sp. or43	Actinobacteria
C14	JACLQY010000057	1	*Streptomyces* sp. TYQ1024	Actinobacteria
C15	WWIC01000086.1	1*	*Streptomyces* sp. SID5643	Actinobacteria
C16	CP012949.1	14	*Streptomyces ambofaciens*	Actinobacteria
C17	CP012382.1	14	*Streptomyces ambofaciens*	Actinobacteria
C18	NZ_CP034587.1	19	*Streptomyces luteoverticillatus*	Actinobacteria
C19	BMTM01000004.1	2	*Streptomyces janthinus*	Actinobacteria
C20	JACHLX010000001	9	*Streptomyces collinus*	Actinobacteria
C21	BMSO01000001.1	1	*Streptomyces bellus*	Actinobacteria
C22	CP023694.1	15	*Streptomyces coeruleorubidus*	Actinobacteria
C23	BMUO01000004.1	3	*Streptomyces violaceochromogenes*	Actinobacteria
C24	BNCB01000007.1	3	*Streptomyces achromogenes* subsp. *rubradiris*	Actinobacteria
C25	RPRR01000011.1	1	*Streptomyces* sp. WAC05858	Actinobacteria
C26	CP007153.2	31	*Streptomyces* sp. AgN23	Actinobacteria
C27	CP022545.1	17	*Streptomyces* sp. 11-1-2	Actinobacteria
C28	NZ_JOJK01000031	1	*Streptomyces mediolani*	Actinobacteria
C29	WWID01000099.1	1	*Streptomyces* sp. SID5614	Actinobacteria
C30	SMKT01000002.1	1	*Actinomadura* sp. KC06	Actinobacteria
C31	NZ_BCQT01000018	3	*Actinomadura macra*	Actinobacteria
C32	BONX01000002.1	1	*Plantactinospora mayteni*	Actinobacteria
C33	CP028159.1	1	*Plantactinospora* sp. BB1	Actinobacteria
C34	NZ_CP028158.1	1	*Plantactinospora* sp. BC1	Actinobacteria
C35	BONW01000039.1	1	*Plantactinospora endophytica*	Actinobacteria
C36	NZ_PJMP01000001	1	*Plantactinospora* sp. CNZ320	Actinobacteria
C37	NZ_SHKQ01000001	1	*Plantactinospora* sp. CNZ321	Actinobacteria
C38	JADBEB010000001	1	*Plantactinospora soyae*	Actinobacteria
C39	QREB01000001.1	25	*Streptomyces* sp. 67	Actinobacteria
C40	VUOB01000045.1	2	*Goodfellowiella* sp. AN110305	Actinobacteria
C41	NZ_MUYM01000003	1	*Lentzea kentuckyensis*	Actinobacteria
C42	NZ_FNFF01000027	1	*Streptomyces indicus*	Actinobacteria
C43	LWLA01000014.1	1	*Streptomyces* sp. CB03911	Actinobacteria
C44	WWJE01000026.1	1	*Streptomyces* sp. SID4936	Actinobacteria
C45	NZ_CP029788.1	7	*Streptomyces actuosus*	Actinobacteria
C46	CP061281.1	12	*Streptomyces* sp. CRXT-Y-14	Actinobacteria
C47	BNBR01000004.1	2	*Streptomyces griseosporeus*	Actinobacteria
C48	NZ_CP026730.1	22	*Streptomyces* sp. CB09001	Actinobacteria
C49	CP025394.1	23	*Kitasatospora* sp. MMS16-BH015	Actinobacteria
C50	BMMP01000010.1	1	*Streptomyces daqingensis*	Actinobacteria

C51	PHUI01000001.1	11	*Streptomyces* sp. 69	Actinobacteria
C52	NZ_BMUT01000017	1	*Streptomyces hiroshimensis*	Actinobacteria
C53	NZ_BMPQ01000032	1	*Streptomyces flaveus*	Actinobacteria
C54	FNTO01000001.1	26	*Streptomyces* sp. 2231.1	Actinobacteria
C55	LIWA01000006.1	1	*Streptomyces* sp. CB01883	Actinobacteria
C56	RJUU01000001.1	3	*Streptomyces* sp. 840.1	Actinobacteria
C57	PJNA01000001.1	25	*Streptomyces* sp. 5112.2	Actinobacteria
C58	RJKP01000002.1	7	*Streptomyces* sp. PanSC9	Actinobacteria
C59	JAAGNI010000261	1	*Streptomyces* sp. SID9727	Actinobacteria
C60	JABERD010000027	1	*Streptomyces* sp. S3(2020)	Actinobacteria
C61	QURH01000180.1	1	*Actinomadura logoneensis*	Actinobacteria
C62	LMWO01000018.1	1	*Streptomyces canus*	Actinobacteria
C63	LMDL01000004.1	1	*Streptomyces* sp. Root369	Actinobacteria
C64	WWGY01000062.1	2	*Streptomyces* sp. SID8350	Actinobacteria
C65	NZ_WWGV01000013	2	*Streptomyces* sp. SID8356	Actinobacteria
C66	WWGX01000064.1	1	*Streptomyces* sp. SID8352	Actinobacteria
C67	WSTF01000006.1	1	*Streptomyces* sp. 196(2019)	Actinobacteria
C68	JAANNR010000001	8	*Streptomyces* sp. KAI-26	Actinobacteria
C69	CP024957.1	24	*Streptomyces cavourensis*	Actinobacteria
C70	JAANOE010000005	2	*Streptomyces* sp. CAI-24	Actinobacteria
C71	WWGS01000014.1	6	*Streptomyces* sp. SID8359	Actinobacteria
C72	FMCC01000165.1	1	*Streptomyces* sp. DvalAA-19	Actinobacteria
C73	NZ_CP011522.1	9	*Streptomyces* sp. CFMR 7	Actinobacteria
C74	FZOD01000012.1	1	*Streptosporangium subroseum*	Actinobacteria
C75	VJZC01000069.1	1	*Streptomyces spongiae*	Actinobacteria
C76	JAFCNB010000001	1	*Microbispora* sp. RL4-1S	Actinobacteria
C77	NZ_BLPG01000001	1	*Phytohabitans rumicis*	Actinobacteria
C78	MRYD01000172.1	1*	*Streptomyces pharetrae*	Actinobacteria
C79	BNBZ01000011.1	3	*Streptomyces zaomyceticus*	Actinobacteria
C80	QFXB01000020.1	2	*Streptomyces* sp. NWU49	Actinobacteria
C81	NZ_AZXH01000039	1	*Salinispora tropica*	Actinobacteria
C82	JAHCSG010000003	1	*Williamsia* sp. CHRR-6	Actinobacteria

Table S5: Primers used for cloning of *crz* genes. Restriction sites are underlined, extra stop codons are marked red.

Name	Application	Sequence (5'→3')
**Crz1.1_NdeI_for**	Cloning of *crz1.1* into pET28a	TGA<u>CATATG</u>CTTGGGACCGACACG
**Crz1.1_HindIII_rev**	Cloning of *crz1.1* into pET28a	TAT<u>AAGCTT</u>TCACAGCAGGGAGAAGGG
**Crz1.2_NdeI_for**	Cloning of *crz1.2* into pET28a	TAT<u>CATATG</u>CTGCGCGACGCGGAC
**Crz1.2_HindIII_rev**	Cloning of *crz1.2* into pET28a	TAT<u>AAGCTT</u>TCATCGCGTCTGCTCCTTC
**Crz2_NdeI_for**	Cloning of *crz2* into pET28a	TGA<u>CATATG</u>GACGCGATGAGTGACACCT
**Crz2_HindIII_rev**	Cloning of *crz2* into pET28a	TAT<u>AAGCTT</u>CTCAAACGCGGGCATGGGA

Supplementary References

[1] T. A. Lundy, S. Mori, S. Garneau-Tsodikova, *RSC Chem. Biol.* 2020, **1**, 233.

[2] M. A. Marahiel, T. Stachelhaus, H. D. Mootz, *Chem. Rev*. 1997, **97**, 2651.

[3] A. M. Gulick, *ACS Chem. Biol.* 2009, **4**, 811.

[4] I. Letunic, P. Bork, *Nucleic Acids Res.* 2021, **49**, W293.

[5] J. B. Patteson, C. M. Fortinez, A. T. Putz, J. Rodriguez-Rivas, L. H. Bryant,III, K. Adhikari, M. Weigt, T. M. Schmeing, B. Li, *J. Am. Chem. Soc.* 2022, **144**, 14057.

[6] B. R. Terlouw, K. Blin, J. C. Navarro-Muñoz, N. E. Avalon, M. G. Chevrette, S. Egbert et al., *Nucleic Acids Res.* 2023 **51**, D603.

[7] A. M. Waterhouse, J. B. Procter, D. M. A. Martin, M. Clamp, G. J. Barton, *Bioinformatics* 2009, **25**, 1189.

[8] R. He, J. Zhang, Y. Shao, S. Gu, C. Song, L. Qian, W.-B. Yin, Z. Li, *PLoS Comput. Biol.* 2023, **19**, e1011100.

[9] C. Rausch, I. Hoof, T. Weber, W. Wohlleben, D. H. Huson, *BMC Evol. Biol.* 2007, **7**, 78.

[10] G. E. Crooks, G. Hon, J.-M. Chandonia, S. E. Brenner, *Genome Res.* 2004, **14**, 1188.

[11] E. C. Meng, T. D. Goddard, E. F. Pettersen, G. S. Couch, Z. J. Pearson, J. H. Morris, T. E. Ferrin, *Protein Sci.* 2023, **32**, e4792

[12] K. Blin, S. Shaw, H. E. Augustijn, Z. L. Reitz, F. Biermann, M. Alanjary, A. Fetter, B. R. Terlouw, W. W. Metcalf, E. J. N. Helfrich, G. P. van Wezel, M. H. Medema, T. Weber, *Nucleic Acids Res.* 2023 **51**, W46.
